# Distinctive Expansion of Potential Virulence Genes in the Genome of the Oomycete Fish Pathogen *Saprolegnia parasitica*

**DOI:** 10.1371/journal.pgen.1003272

**Published:** 2013-06-13

**Authors:** Rays H. Y. Jiang, Irene de Bruijn, Brian J. Haas, Rodrigo Belmonte, Lars Löbach, James Christie, Guido van den Ackerveken, Arnaud Bottin, Vincent Bulone, Sara M. Díaz-Moreno, Bernard Dumas, Lin Fan, Elodie Gaulin, Francine Govers, Laura J. Grenville-Briggs, Neil R. Horner, Joshua Z. Levin, Marco Mammella, Harold J. G. Meijer, Paul Morris, Chad Nusbaum, Stan Oome, Andrew J. Phillips, David van Rooyen, Elzbieta Rzeszutek, Marcia Saraiva, Chris J. Secombes, Michael F. Seidl, Berend Snel, Joost H. M. Stassen, Sean Sykes, Sucheta Tripathy, Herbert van den Berg, Julio C. Vega-Arreguin, Stephan Wawra, Sarah K. Young, Qiandong Zeng, Javier Dieguez-Uribeondo, Carsten Russ, Brett M. Tyler, Pieter van West

**Affiliations:** 1 Broad Institute of MIT and Harvard, Cambridge, Massachusetts, United States of America; 2 Aberdeen Oomycete Laboratory, School of Medical Sciences, University of Aberdeen, Aberdeen, United Kingdom; 3 Scottish Fish Immunology Research Centre, School of Biological Sciences, University of Aberdeen, Aberdeen, United Kingdom; 4 Plant-Microbe Interactions, Department of Biology, Utrecht University, Utrecht, The Netherlands; 5 Université de Toulouse; UPS; Laboratoire de Recherche en Sciences Végétales, Castanet-Tolosan, France and CNRS, Laboratoire de Recherche en Sciences Végétales, Auzeville, Castanet-Tolosan, France; 6 Division of Glycoscience, School of Biotechnology, Royal Institute of Technology (KTH), AlbaNova University Centre, Stockholm, Sweden; 7 Laboratory of Phytopathology, Wageningen University, Wageningen, The Netherlands; 8 Centre for BioSystems Genomics, Wageningen, The Netherlands; 9 Dipartimento di Gestione dei Sistemi Agrari e Forestali, Università degli Studi Mediterranea, Reggio Calabria, Italy; 10 Department of Biological Sciences, Bowling Green State University, Bowling Green, Ohio, United States of America; 11 Theoretical Biology and Bioinformatics, Department of Biology, Utrecht University, Utrecht, The Netherlands; 12 Virginia Bioinformatics Institute, Virginia Polytechnic Institute and State University, Blacksburg, Virginia, United States of America; 13 ENES Unidad León, Universidad Nacional Autónoma de México, León, Mexico; 14 Departamento de Micología, Real Jardín Botánico CSIC, Madrid, Spain; Virginia Tech, United States of America

## Abstract

Oomycetes in the class Saprolegniomycetidae of the Eukaryotic kingdom Stramenopila have evolved as severe pathogens of amphibians, crustaceans, fish and insects, resulting in major losses in aquaculture and damage to aquatic ecosystems. We have sequenced the 63 Mb genome of the fresh water fish pathogen, *Saprolegnia parasitica.* Approximately 1/3 of the assembled genome exhibits loss of heterozygosity, indicating an efficient mechanism for revealing new variation. Comparison of *S. parasitica* with plant pathogenic oomycetes suggests that during evolution the host cellular environment has driven distinct patterns of gene expansion and loss in the genomes of plant and animal pathogens. *S. parasitica* possesses one of the largest repertoires of proteases (270) among eukaryotes that are deployed in waves at different points during infection as determined from RNA-Seq data. In contrast, despite being capable of living saprotrophically, parasitism has led to loss of inorganic nitrogen and sulfur assimilation pathways, strikingly similar to losses in obligate plant pathogenic oomycetes and fungi. The large gene families that are hallmarks of plant pathogenic oomycetes such as *Phytophthora* appear to be lacking in *S. parasitica*, including those encoding RXLR effectors, Crinkler's, and Necrosis Inducing-Like Proteins (NLP). *S. parasitica* also has a very large kinome of 543 kinases, 10% of which is induced upon infection. Moreover, *S. parasitica* encodes several genes typical of animals or animal-pathogens and lacking from other oomycetes, including disintegrins and galactose-binding lectins, whose expression and evolutionary origins implicate horizontal gene transfer in the evolution of animal pathogenesis in *S. parasitica*.

## Author Summary

Fish are an increasingly important source of animal protein globally, with aquaculture production rising dramatically over the past decade. *Saprolegnia* is a fungal-like oomycete and one of the most destructive fish pathogens, causing millions of dollars in losses to the aquaculture industry annually. *Saprolegnia* has also been linked to a worldwide decline in wild fish and amphibian populations. Here we describe the genome sequence of the first animal pathogenic oomycete and compare the genome content with the available plant pathogenic oomycetes. We found that *Saprolegnia* lacks the large effector families that are hallmarks of plant pathogenic oomycetes, showing evolutionary adaptation to the host. Moreover, *Saprolegnia* harbors pathogenesis-related genes that were derived by lateral gene transfer from the host and other animal pathogens. The retrotransposon LINE family also appears to be acquired from animal lineages. By transcriptome analysis we show a high rate of allelic variation, which reveals rapidly evolving genes and potentially adaptive evolutionary mechanisms coupled to selective pressures exerted by the animal host. The genome and transcriptome data, as well as subsequent biochemical analyses, provided us with insight in the disease process of *Saprolegnia* at a molecular and cellular level, providing us with targets for sustainable control of *Saprolegnia*.

## Introduction

*Saprolegnia* species are watermolds or oomycetes that are endemic to probably all fresh water ecosystems. These understudied pathogens can cause destructive diseases of amphibians, crustaceans, fish and insects in aquaculture and in natural environments worldwide [1], [2]. With the rise of fish as a principal source of animal protein, and the decline of wild fish stocks, aquaculture production has increased on average by 11% per year worldwide over the past ten years (FAO Fishery Information). Intensive aquatic farming practices have resulted in explosive growth in pathogen populations, which has been exacerbated by the ban of malachite green as a pesticide. Losses due to microbial, parasitic and viral infections are the largest problem in fish farms nowadays, and have a significant effect on animal welfare and sustainability of the industry. The salmon farming industry is particularly affected by *Saprolegnia parasitica*. This pathogen causes Saprolegniosis (also known as Saprolegniasis), a disease characterized by visible grey or white patches of mycelium on skin and fins, and subsequent penetration of mycelium into muscles and blood vessels [1], [3]. It is estimated that 10% of all hatched salmon succumb to *Saprolegnia* infections and losses are estimated at tens of millions of dollars annually [2]. In addition to the damage to the aquaculture industry, the declines of natural salmonid populations have also been attributed to *Saprolegnia* infections [1]. More in-depth knowledge of the epidemiology, biology and pathology of the pathogen is urgently needed. A draft genome sequence of *S. parasitica* provides an excellent starting point to investigate the disease process at the molecular and cellular level and may lead to novel avenues for sustainable control of Saprolegniosis.

**Figure 1:**
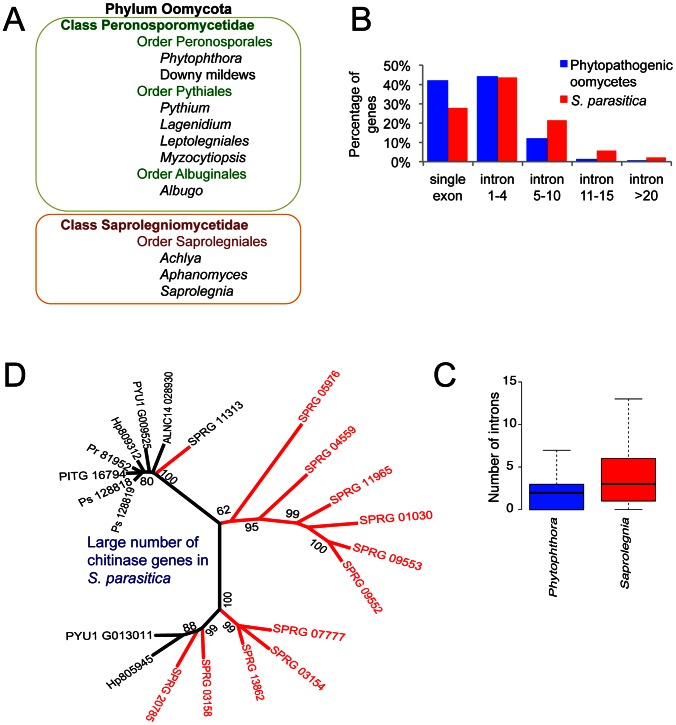
Taxonomy and ancestral genomic features in *S. parasitica*. *(A)* Animal pathogenic and plant pathogenic oomycetes reside in different taxonomic units. *(B)* Comparison of intron number in phytopathogenic oomycetes (the average count from the total genes of *P. infestans*, *P. ramorum*, *P. sojae*, *Py. ultimum* and *H. arabidopsidis*) and *S. parasitica* among all genes. *(C)* Significant difference in intron number in 4008 orthologous genes shared by *S. parasitica* and *Phytophthora* species (average intron count of *P. infestans*, *P. sojae* and *P. ramorum*). (Wilcoxon test, p<0.001). *(D)* Large number of chitinase genes belonging to CAZy family GH-18 in *S. parasitica* (red) compared to other oomycetes (black; Ps = *P. sojae*, Pr = *P. ramorum*, PITG = *P. infestans*, Hp = *H. arabidopsidis*, Pyu = *Py. ultimum*, ALNC = *A. laibachii*). The phylogenetic tree was constructed with chitinase genes from oomycetes using Maximum likelihood method.

Animal pathogens have evolved independently multiple times in lineages such as Stramenopila, Alveolata, Amebozoa, Euglenozoa and Mycota, as well as in numerous bacterial lineages. Oomycetes such as *Saprolegnia* belong to the kingdom Stramenopila (Patterson, 1989), syn. Straminipila (Dick, 2001), that includes photosynthetic algae such as kelp and diatoms, ubiquitous saprotrophic flagellates such as *Cafeteria roenbergensis*, and obligate mammalian parasites such as *Blastocystis* [4], [5]. Although many *Saprolegnia* and related species are capable of causing diseases on a wide range of animal hosts including humans, relatively little is known about their mechanisms of pathogenicity. Among the oomycetes, most animal pathogens including *S. parasitica* belong to the class Saprolegniomycetidae (Figure 1A). The oomycetes also include many plant pathogens and these are mainly concentrated within the class Peronosporomycetidae. There are a small number of interesting exceptions to this otherwise sharp dichotomy, including the mammalian pathogen *Pythium insidiosum* (Peronosporomycetidae) and the plant pathogens *Aphanomyces euteiches* and *Aphanomyces cochlioides* (Saprolegniomycetidae) [1], [6], [7].

Plant pathogens in the class Peronosporomycetidae include *Phytophthora* and *Pythium* species, downy mildew pathogens and white rusts (Figure 1A). Well-known examples are the potato late blight pathogen *Phytophthora infestans* that caused the great Irish famine in the 1840s [8], the soybean root rot pathogen *Phytophthora sojae* [9], and the sudden oak death pathogen *Phytophthora ramorum* [10]. Sequenced and assembled genomes have been generated for *P. sojae and P. ramorum* [11], *P. infestans* [12], and for several relatives including the broad host range plant pathogens *Phytophthora capsici* [13] and *Pythium ultimum* [14], the downy mildew pathogen of *Arabidopsis*, *Hyaloperonospora arabidopsidis* [15], and the white blister rust of Brassicaceae, *Albugo candida* [16]. Genome analyses have revealed a bi-partite genome organization in these pathogens, in which gene dense regions containing clusters of orthologs with well-conserved sequences and gene order are interspersed with repeat-rich regions containing rapidly evolving families of virulence genes and numerous transposons [12], [17], [18].

The virulence gene families of plant pathogenic oomycetes encode numerous hydrolytic enzymes for degradation of plant carbohydrates, extracellular toxins such as NLP and PcF toxins, and at least three families of cell-entering effector proteins, RXLR effectors, CHXC effectors and Crinkler proteins [7], [11], [19], [20]. These classes of effector proteins contain amino acid sequence motifs (RXLR, CHXC, and LFLAK respectively) that are involved in entry into the host plant cell [12], [21], [22], [23], [24], [25] and effector domains that target diverse host physiological processes to suppress immunity and promote infection [17]. In the genome of the broad host-range necrotrophic oomycete *Py. ultimum*, which primarily targets immature or stressed plant tissues, enzyme families are expanded that enable degradation of readily accessible carbohydrates such as pectins, starch, and sucrose, while RXLR effectors appear to be completely absent [14]. In the obligate biotroph *H. arabidopsidis*, most gene families are smaller, especially those encoding hydrolytic enzymes [15]. Interestingly, EST libraries from the saprolegniomycete plant pathogen *A. euteiches* revealed the presence of Crinkler effectors (but neither NLP toxins nor RXLR effectors) [26], suggesting that Crinklers are ancestral to oomycete pathogens [23].

So far, only limited genomic resources are available for animal pathogenic oomycetes. Analysis of small sets of EST data of *S. parasitica* [27], [28] and *Py. insidiosum* [29], revealed the presence of secreted protein families with potential roles in virulence such as glycosyl hydrolases, proteases, and protease inhibitors, as well as proteins involved in protection against oxidative stress. The *S. parasitica* data set included a host-targeting protein SpHtp1 (*S. parasitica* host-targeting protein 1) that was subsequently demonstrated to enter fish cells through binding to a tyrosine-O-sulfated fish cell surface ligand [30].

Here we report the genome sequence and transcriptome analysis of *S. parasitica*, the first genome sequenced from an animal pathogenic oomycete. We compared the genome to those of plant pathogenic oomycetes, revealing distinctive genome expansions and adaptations tailored to the physiology of the respective hosts. This study adds to our understanding of mechanisms for invasion and colonization of animal host cells by eukaryotic pathogens.

## Results

### A compact, highly polymorphic genome exhibiting extensive loss of heterozygosity

The strategy of whole genome shotgun sequencing was applied to *S. parasitica* strain CBS223.65, which is a strain isolated from infected pike (*Esox lucius*). A combination of 454 (fragment and 3 kb jumping libraries) and Sanger (Fosmid library) sequencing data (∼50-fold average read coverage) were used to assemble the genome, yielding an initial assembly length of 53 Mb and a scaffold N50 length of 281 kb (see Table S1 for additional assembly statistics). Read coverage and rates of polymorphism were computed based on alignments of Illumina data (generated for polymorphism discovery) to the genome assembly. Based on the distribution of coverage and polymorphisms (Figure S1A and S1B), we conclude that the assembly represents a composition of regions of diploid consensus (76% of the assembly) with the remainder corresponding to separately assembled haplotypes, resulting from the high polymorphism rate with a peak of 2.6% (Table S2 and Figure S1C). Taking into account the 24% of the assembly corresponding to separately-assembled haplotypes, the total assembled haploid genome size was adjusted to 42.3 Mb (see Text S1). Based on read coverage we estimated the total genome size to be 62.8 Mb. The remaining 20.4 Mb of genomic sequence were estimated to correspond to collapsed tandem repeat content in the genome assembly (Text S1). Read coverage analysis of the separated haplotypes and of regions showing loss of heterozygosity (see below) produced an overall genome size estimate of 63 Mb (Text S1), consistent with our effective assembled genome size estimate. The difference between the assembly size and the read coverage estimate likely results from tandem repeats collapsed in the assembly and uncertainties in read alignments, as observed in other oomycete genome sequence assemblies (Table 1). More than 98% of Trinity [31] *de novo* assembled transcripts from *in vitro* growth RNA-Seq data (described in Material and Methods) mapped to the genome assembly, indicating that the expressed gene content is very well represented by the assembled genome. The genome size of *S. parasitica* is consistent with the size range of most previously sequenced oomycete genomes, which rank from 45 Mb (*Py. ultimum*) [14] to 65 Mb (*P. ramorum*) [11], far below the outlier of 240 Mb (*P. infestans*) [12] (Table 1).

**Table 1:** Genome statistics and intron features of oomycetes.

	*Saprolegnia parasitica*	*Phytophthora infestans*	*Phytophthora sojae*	*Phytophthora ramorum*	*Hyaloperonospora arabidopsidis*	*Pythium ultimum*
Estimated genome size	63 Mb	240 Mb	95 Mb	65 Mb	99 Mb	45 Mb
Total contig length	42.3 Mb^a^	190 Mb^b^	78 Mb^b^	54 Mb^b^	82 Mb^b^	45 Mb^b^
G+C content	58%	51%	54%	54%	47%	52%
Repeat (%)^c^	40% (17%)^d^	74%	39%	28%	35%	7%
Number of genes	17,065	17,797	16,988	14,451	14,567	15,291
Gene density kb/per gene	2.6	10.7	4.6	3.7	5.6	2.9
Gene Length mean	1521 bp	1525 bp	1614 bp	1624 bp	1113 bp	1503 bp
Genes with introns	73%	67%	56%	53%	49%	62%
Mean exon number per gene	4	2.8	2.6	2.6	2.0	2.6
Exon length mean	337 bp	475 bp	536 bp	552 bp	493 bp	502 bp
Intron length mean	75 bp	125 bp	124 bp	123 bp	150 bp	121 bp

a Total contig length adjusted for the regions of haplotype assemblies.

b Genome statistics derived from publication of these genomes.

c Measured by repeatMasker with de novo RepeatScout.

d The repeat content in the assembled sequence is listed in the brackets.

Approximately one-third of the assembled *S. parasitica* genome was found to correspond to regions exhibiting loss of heterozygosity (LOH) (Figure S1C and Text S1). LOH resulting from mitotic instability has been observed in other oomycete genomes [13], [32], [33], and provides a potential adaptive mechanism that promotes expression of genetic diversity within a clonal pathogen population [13]. The prevalence of LOH within a *S. parasitica* population and the role LOH plays in *S. parasitica* evolution remain to be determined.

A total of 20,113 coding gene models were computationally predicted, with 3,048 pairs of coding genes assigned as alleles within separately assembled haplotype regions (Table S3), yielding an adjusted coding gene count of 17,065, similar in magnitude to counts of genes identified in other sequenced oomycete genomes (Table 1). There are 5,291 coding genes (31% of total) found to reside within regions of LOH (Table S3). No obvious enrichment or depletion of biologically relevant gene functions could be detected within the defined regions of LOH that would suggest that the LOH observed had resulted from selection in the individual strain sequenced.

The gene density found in *S. parasitica* is one of the highest reported so far for oomycetes, with one gene per 2.6 kb (Table 1). This is slightly denser than in *Py. ultimum* (one gene per 2.9 kb) and *A. candida* (2.9 kb), and much denser than in *P. infestans* (one gene per 10.7 kb). Many of the *S. parasitica* genes are novel; only 40% of the *S. parasitica* predicted proteins have homologs with more than 50% sequence similarity to those from other organisms, including oomycetes. A conserved core-proteome of 4,215 proteins can be identified for plant pathogenic peronosporomycetes based on genomes from multiple *Phytophthora* species, *H. arabidopsidis* and *Py. ultimum* [14]. Of these 4215, *S. parasitica* shares only 3518 orthologs (Figure S2A), of which only about 40% show strong sequence similarity (>50%). 20% of the core set is not detectable in the *S. parasitica* proteome (Figure S2B). Although the genomes of the peronosporomycetes show substantial conservation of gene order (synteny), little of this synteny is preserved in *S. parasitica*, as was observed for *A. candida* [16].

Interestingly, compared to other oomycetes, *S. parasitica* genes contain a larger number of introns (Figure 1B, Table 1). More than 73% of the *S. parasitica* genes contain at least one intron, compared to 50–60% in other oomycete species (Table 1). Among 4008 orthologs shared between *S. parasitica* and three *Phytophthora* species, the majority of the genes have different numbers of introns. For example, more than half of the *S. parasitica* genes have more introns than their orthologs in *Phytophthora*, and 15% of the *S. parasitica* genes have 5 or more additional exons compared to their *Phytophthora* orthologs (Figure 1C). The intron abundance in *S. parasitica* potentially more closely matches the ancestral state, assuming a trend of intron reduction as found in animal and fungal lineages [34].

The *S. parasitica* genome has very few known mobile elements, which is consistent with its smaller size compared to the transposon-rich *Phytophthora* genomes. Of the 160 repeat families identified among all sequenced *Phytophthora* species, only one LTR retrotransposon family was found in the *S. parasitica* genome (Figure S3A). This group of LTR elements, which occur at low copy numbers (<20) in known oomycete genomes (Figure S3B), is thus ancient. The largest transposon family in *S. parasitica* (approx. 50 copies in the assembled sequence, and an estimated a few hundred copies in the genome) belongs to the LINE repeat group (Text S1). LINE elements are abundant in animal genomes and play roles in genome evolution and modulation of gene expression [35]. Curiously, the *S. parasitica* LINE element family is absent from the *Phytophthora* genomes but shares sequence similarity with the LINE elements from animal genomes (Figure S3C), raising the possibility that this family was acquired from an animal host.

### *Saprolegnia parasitica* has a very large kinome

Eukaryotic protein kinases (ePKs) regulate a myriad of cellular activities by phosphorylating target proteins in response to internal or external signals. *S. parasitica* has one of the largest kinomes that have been identified to date, with 543 predicted ePKs. For example, *S. parasitica* has 65 more ePKs than the human kinome by the same prediction criteria (Figure S4A). Eukaryotic protein kinases have been classified into eight major groups based on sequence similarity. We classified members of the *S. parasitica* ePK superfamily using a set of HMMs based on previously identified kinases (Figure S4A). *S. parasitica* has a large expansion of tyrosine kinase-like proteins with 298 members and a large number of unclassified kinases (114), suggesting novel functions performed by the *S. parasitica* kinome. Interestingly, the *S. parasitica* kinome contains several kinase families that have typically not been seen outside the metazoan clade, for example CAMK2, NUAK, SNRK, and PHK from the Ca^2+^ calmodulin-dependent kinase group. Some of these “metazoan” kinases are shared with plant pathogenic oomycetes [36], substantially pushing back the evolutionary origin of these kinases. We found 131 kinases containing predicted transmembrane helices (Figure S4B), suggesting that *S. parasitica* has a large number of protein kinases that may function as cell surface receptors with roles in signaling. Such a large receptor repertoire may facilitate the recognition of stimuli from extracellular abiotic and biotic environments.

### Chitin metabolism

The cell wall of a pathogen plays a central role at the host-pathogen interface. In particular, cell wall related proteins and polysaccharides are a large source of PAMPs (Pathogen Associated Molecular Patterns) of both animal and plant pathogens [37], [38]. In addition, cell wall carbohydrate biosynthetic enzymes represent a potential target of antimicrobial compounds when similar activities are not encountered in the host. This is illustrated by the demonstration that the specific inhibition of chitin synthase (CHS) in *Saprolegnia* leads to cell death although chitin represents no more than 1% of the total cell wall carbohydrates of the pathogen [39], [40].Chitin is a structural crystalline polymer typically associated with the fungal cell wall. Historically, the absence of chitin in *Phytophthora* [41] has led to the general concept that oomycetes are devoid of chitin and that cellulose, the major load-bearing structural polysaccharide in oomycete walls, is a key feature distinguishing oomycetes from true fungi. However, the occurrence of chitin has since been demonstrated in various oomycete species belonging to the Saprolegniales [39], [40], [42]. In addition, GlcNAc-based carbohydrates that do not seem to correspond to crystalline chitin, but whose biosynthesis is most likely performed by putative chitin synthase gene products are present in the walls of *Aphanomyces euteiches* [43].The *S. parasitica* genome appears to contain genes that encode enzymes involved in chitin biosynthesis, modification and degradation (Table S4). Out of these, six genes correspond to putative chitin synthases (SPRG_02074, SPRG_02554, SPRG_04151, SPRG_09812, SPRG_06131 and SPRG_19383). Interestingly, this number is higher than in other oomycetes where only one or two CHS putative genes have been detected [39], [43] (Table S4). The only oomycete CHS gene product for which the function has been unequivocally demonstrated through heterologous expression and *in vitro* biochemical assays is CHS2 from *Saprolegnia monoica* [39]. Thus, as for most CHS genes from other oomycetes, the function of the *S. parasitica* genes annotated here as CHS remains to be demonstrated. As reported earlier in *S. monoica* [39], two of the newly identified *S. parasitica* CHS gene products (SPRG_09812 and SPRG_04151) contain a so-called MIT (Microtubule Interacting and Trafficking (or Transport)) domain. These MIT domains are possibly involved in the trafficking and delivery of the corresponding CHS at the apex of the hyphal cells, as previously suggested for *S. monoica* [39]. There are also a large number (14) of chitinase genes in *S. parasitica* compared to other oomycetes, which could represent many ancestral forms of oomycete chitinases (Figure 1D).

### Adaptations of metabolism to animal pathogenesis

There are major structural and physiological differences between plant and animal cells, and thus the metabolisms of plant and animal pathogens have likely adapted accordingly to the respective host cellular environments. Pectin is a major constituent of plant cell walls and a target for extracellular enzymes produced by pathogenic and saprophytic microorganisms. Plant pathogenic fungi and oomycetes produce a large array of enzymes to degrade pectin, including polygalacturonase (PG), pectin and pectate lyases (PL), and pectin methylesterases (PME). Animal cells lack a cell wall, and as might be expected, the pathogen *S. parasitica* encodes very few cell wall degrading enzymes in its genome. Genes encoding hydrolytic enzymes such as cutinase and pectin methyl esterases appear to be absent, and PL and PG genes are greatly reduced in numbers as compared to plant pathogenic oomycetes (Table 2). The remaining small numbers of PLs and PGs may play a role in the saprophytic life stage of *S. parasitica* in the aquatic environment outside of fish hosts.

**Table 2:** Gene families potentially involved in pathogenesis in *Saprolegnia parasitica*.

Gene families^a^	Spa	Pinf	Psoj	Pram	Hpa	Pult	Aph^b^
**RXLR**	0	563	396	374	134	0	
**Crinklers**	0	196	100	19	20	26	+
**NPP1-like proteins**	0	27	39	59	10	7	
**Elicitin and elicitin like**	29	50	66	60	17	45	
**Cutinase**	0	4	16	4	2	0	
**Pectin methyl esterases**	0	11	19	13	3	0	
**Glycosyl hydrolase**	74	147	183	162	53	66	
**Pectate lyases**	0	33	20	24	6	14	
**Polygalacturonases**	2	23	25	16	3	2	
**PAN** c	13	11	8	15	2	17	
**CBEL** c	40	4	5	5	0	3	+
**Ricin** c	40	6	11	11	3	4	
**Gal-Lectin-binding** c	5	0	0	0	0	0	
**Jacalin-like lectin** c	0	4	4	4	0	0	
**Legume-like lectin** c	3	3	3	3	3	3	
**Disintegrin** c	16	0	0	0	0	0	
**Protease inhibitors, all**	7	34	24	16	3	15	
**Proteases, all**	270	194	185	190	143	200	+
**Serine proteases**	76	45	32	41	34	34	
**Metalloproteases**	69	51	50	49	48	51	
**Cysteine proteases**	85	67	71	69	33	77	
**ABC transporter, all**	129	161	186	183	55	136	+
**Kinases**	543	444	436	432	271	166	+
**Notch protein**	15	1	1	1	1	1	
**Haemolysin E**	9	0	0	0	0	0	

a Spa = *Saprolegnia parasitica*, Pinf = *Phytophthora infestans*, Psoj = *P. sojae*, Pram = *P. ramorum*, Hpa = *Hyaloperonospora arabidopsidis*, Pult = *Pythium ultimum*, Aph = *Aphanomyces euteiches*.

b The presence of the protein families were searched in the *Aphanomycete* EST database by BLASTP. A positive hit (E value<1e-8 sequence similarity >30%) is indicated by +.

c Lectin and lectin-like families.

**Figure 2:**
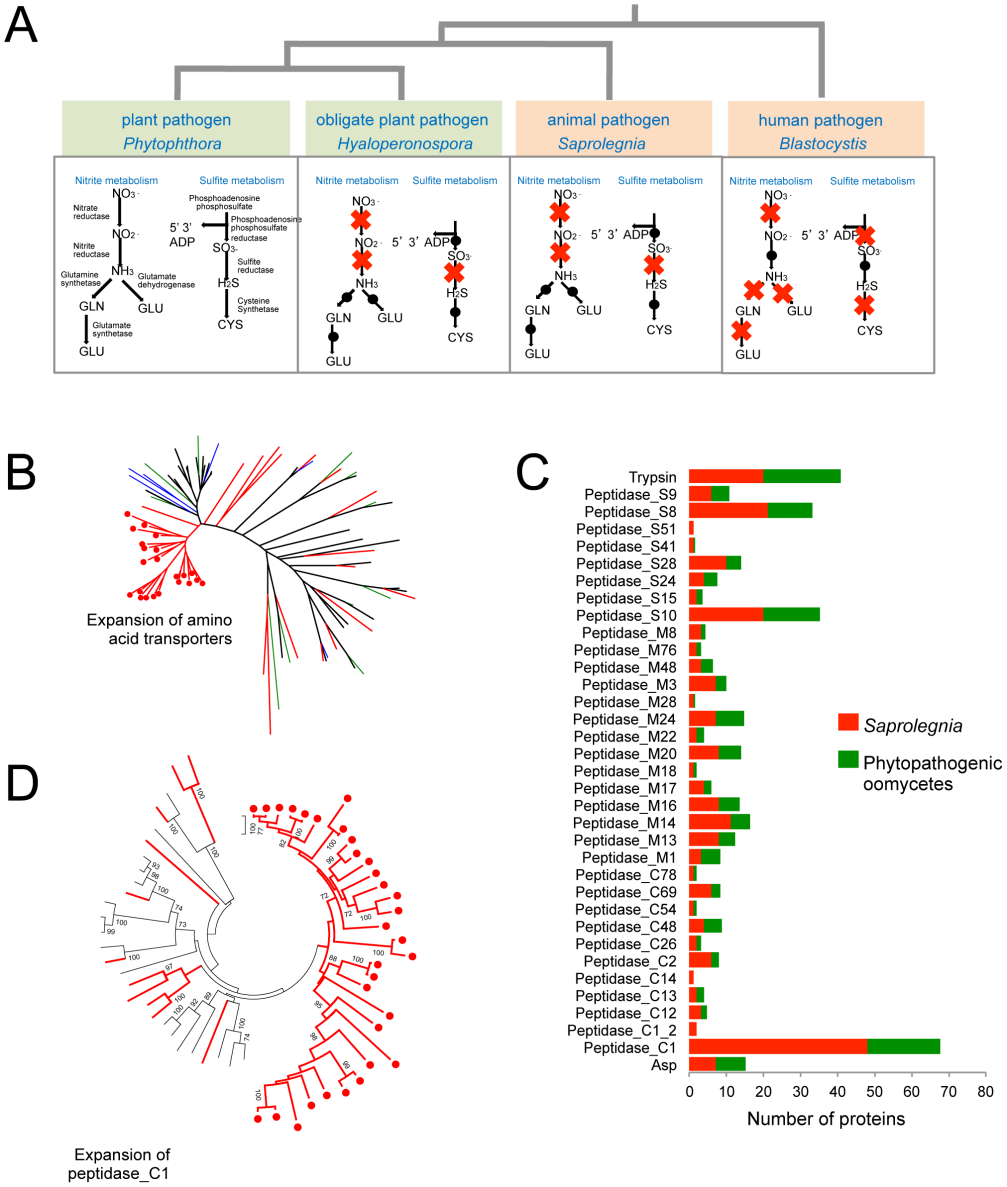
Metabolic adaptations to animal pathogenesis. *(A)* Independent degeneration of nitrite and sulfite metabolic pathways in animal pathogens and obligate biotrophic plant pathogens. Red cross indicates the gene encoding the enzyme is absent in the genome. *(B)* Lineage specific expansion of amino acid transporters. Members from *Pythium* (black), *Hyaloperonospora* (green), *Albugo* (blue) and *S. parasitica* (red) are included. - The *S. parasitica*-specific clade is marked with red dots. *(C)* Secreted peptidase families in *S. parasitica* and phytopathogenic oomycetes (the average count from the total peptidase genes of *P. infestans*, *P. ramorum*, *P. sojae*, *Py. ultimum* and *H. peronospora*) . Peptidase_C1, Peptidase_S8 and Peptidase_S10 are the largest families in *S. parasitica*. *(D)* Lineage-specific expansion of peptidase_C1 family. Members from *P. sojae*, *P. ramorum* and *P. infestans* (black) and *S. parasitica* (red) are included. The *S. parasitica*-specific clade is marked with red dots.

*S. parasitica* proliferates in host tissue rich in proteins and ammonium. Concomitantly, its pathways involved in inorganic nitrogen and sulfur assimilation have degenerated (Figure 2A). The loss of these metabolic capabilities has occurred independently in the obligate oomycete plant pathogen *H. arabidopsidis* [15], as well as within several lineages of obligate fungal plant pathogens, presumably due to the high level of parasitic adaptation in these organisms. Strikingly the same physical clusters of genes have been lost in each lineage, namely the genes encoding nitrate reductase, nitrite reductase, sulfite reductase and nitrate transporters (Table S5). Also in line with a protein-rich environment that is a major source of both carbon and nitrogen, the *S. parasitica* genome contains 56 genes predicted to encode amino acid transporters. Most of the *S. parasitica* transporters appear to be novel because less than 20 of the predicted amino acid transporters have orthologs or closely related paralogs in other oomycete genomes. Phylogenetic analysis shows there are lineage-specific expansions of amino acid transporter genes in the different oomycete genomes, with recently duplicated *S. parasitica* genes forming the largest group (Figure 2B).

### Other metabolic differences with oomycete plant pathogens

The gene for phospholipase C (PLC) is absent in all of the sequenced peronosporomycete plant pathogens, but is present in *S. parasitica* (SPRG_04373). Phylogenetic analysis groups the *S. parasitica* PLC gene with that of other heterokont species (Figure S5, Table S6). This shows that the *S. parasitica* PLC is most likely to be ancestral and that the absence of PLC in other oomycetes is due to gene loss.

Peronosporomycete plant pathogens are sterol auxotrophs and their genomes are missing most genes encoding enzymes involved in sterol biosynthesis [44]. In contrast, analysis of the EST collection from *A. euteiches* and the *S. parasitica* genome predicts the existence of enzymes that function in a novel sterol biosynthetic pathway [26] which has been shown to lead to the synthesis of fucosterol in *A. euteiches* [45]. Importantly, one of the genes SPRG_09493 encodes a CYP51 sterol-demethylase (Figure S6), a major target of antifungal chemicals that could perhaps also be used to combat Saprolegniomycetes.

### Candidate virulence proteins

Like plant pathogens, *S. parasitica* presumably secretes a battery of virulence proteins to promote infection. Due to co-evolution with the host, virulence proteins are typically rapidly evolving and may appear to be unique to the species, or encoded by recently expanded gene families [17], [19]. The *S. parasitica* genome contains a large number of genes (11,825) that are not orthologous to any known genes in other species (Figure S2A and S2B), and many recently expanded gene families. There are at least 87 pfam domains that are either unique or show recent expansions in *S. parasitica* as compared to other oomycete species (Table S7). An estimated 970 proteins (Table S8) were predicted to be extracellular based on previously established bioinformatics criteria [11], [12], such as the presence of a eukaryotic signal peptide, and lack of targeting signals to organelles or membranes. Many of the expanded families appear to function at the exterior or cell surface of the pathogens, such as proteins containing CBM1 (Carbohydrate Binding Module Family I according to the CAZy database (http://www.cazy.org/; [46]), ricin B lectin, Notch domains, and also numerous peptidases. Among the proteins that are unique to *S. parasitica* compared to plant pathogenic oomycetes, the largest families have similarities to animal-pathogenesis-associated proteins, such as disintegrins, ricin-like galactose-binding lectins and bacterial toxin-like proteins (haemolysin E).

Oomycetes contain an unusually large number of proteins with novel domain combinations, recruited from common metabolic, regulatory and signaling domains [47], [48]. *S. parasitica* contains in total 169 novel domain combinations that are specific to this pathogen (Table S9). As described above, some of the lineage-expanded domains such as CBM and ricin are used for novel combinations to form composite proteins. Additional domains used for novel combinations are the cytochrome p450 and tyrosinase domains. Proteins carrying *S. parasitica*-specific domain combinations are significantly enriched (hypergeometric test, p<0.001) in predicted secreted proteins (3.6% of secretome), whereas only 1.2% of total proteins have *S. parasitica*-specific novel combinations. The enrichment in secreted proteins is strongly suggestive of a role for the novel domain combinations in pathogenesis.

**Figure 3:**
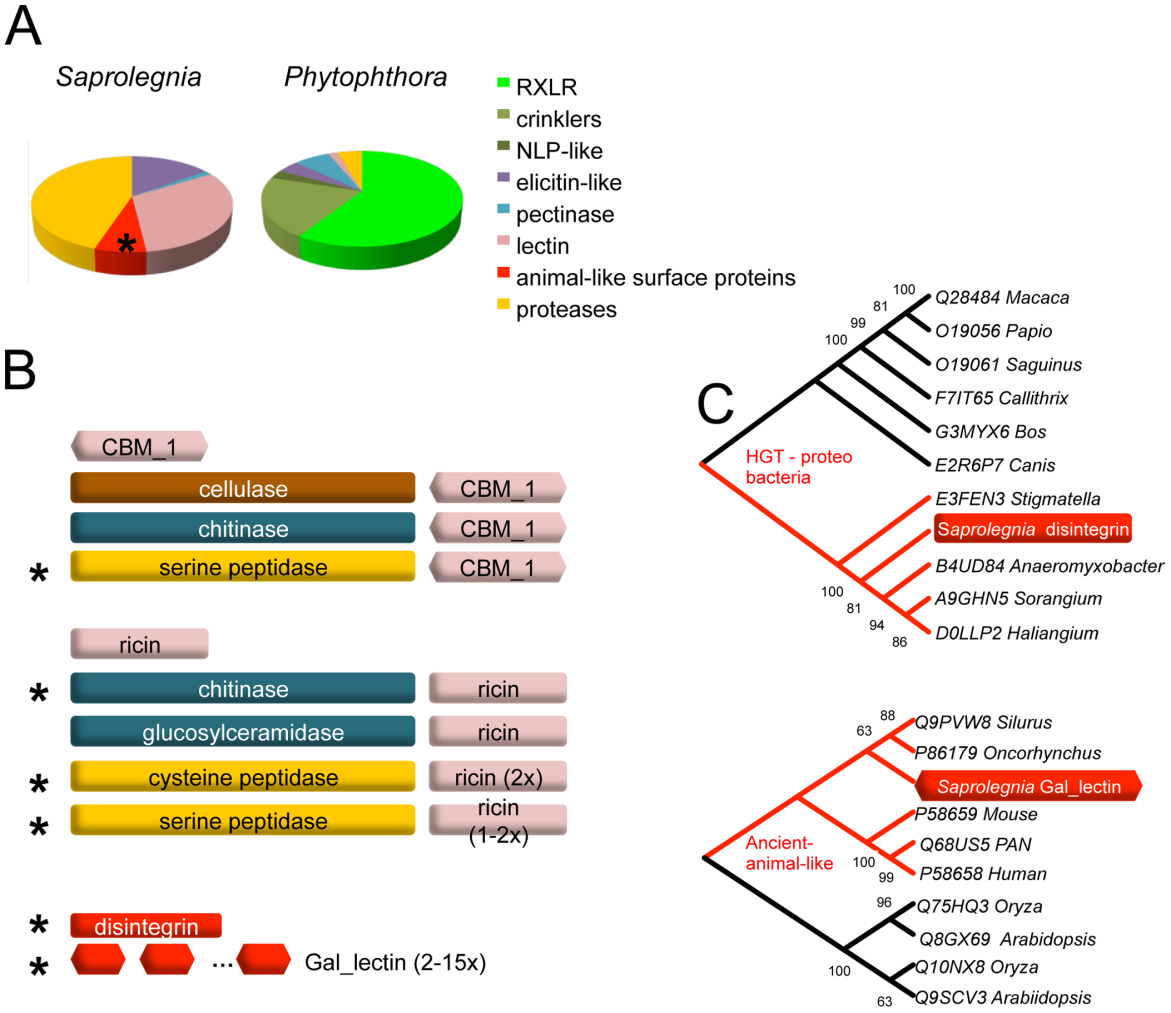
Specialized proteins in the secretome of *S. parasitica*. *(A)* Distributions of major classes of specialized secreted proteins compared between animal and plant pathogenic oomycetes. *P. infestans* represents *Phytophthora* species. *(B) S. parasitica* secreted proteins that carry various lectin domain fusions are schematically drawn. Domains or domain architectures unique to *S. parasitica* are marked with an asterisk. Proteins containing single domains are also listed. *(C)* Phylogenetic relationship of lectins. The *S. parasitica* disintegrin gene (SPRG_01285 groups with bacterial homologs; gal_lectin gene (SPRG_05731)) groups with animal species. All other paralogous *S. parasitica* disintegrin and gal_lectin genes group closely with these two representatives, respectively, and are not shown.

There are about 1000 proteins that are predicted to be secreted by *S. parasitica*, based on criteria used for secretome prediction in other oomycetes [11], [12]. Two groups of proteins dominate the secretome of *S. parasitica:* proteases and lectins (Figure 3A, Table S8). There are over a hundred members in the each of the two groups. In the proteome, *S. parasitica* has one of the largest repertoires of proteases (270) known to date compared to most other single cell or filamentous eukaryotic pathogens (such as most sequenced fungi species) that typically have between 70 and 150 proteases. In almost every family of proteases, including cysteine-, serine- and metallo-proteases (Table 2), there is an expansion in *S. parasitica* compared to *P. sojae* (Figure 2C). The most relatively abundant family of proteases is the papain-like peptidase_C1 proteins, comprising 48 proteins; the other oomycetes contain only about 20 proteins. The majority of papain-like peptidase_C1 genes (80%) have been generated by *S. parasitica* lineage-specific gene duplications and form a lineage-specific clade in the phylogenetic reconstruction (Figure 2D). Amongst the cell-surface associated proteins, ricin_B_lectin-like proteins and CBM1 domain-containing proteins are most abundant (Table 2). There are 40 ricin-like and 40 CBM1 genes in *S. parasitica*, a large expansion compared to other known oomycetes. In some cases, these domains are fused to other secreted protein domains having catalytic activities (protease and cellulose) to form novel proteins unique to oomycetes (Figure 3B). However, typical peronosporomycete proteins were not found, such as CBEL (Cellulose Binding Elicitor Lectin), which contains a CBM1-PAN domain association and mediates the binding of mycelium to cellulosic substrates [49]. The saprolegniomycete plant pathogen *Aphanomyces euteiches* also lacked CBEL proteins [26].

### Protease activities secreted by *Saprolegnia parasitica*

**Figure 4:**
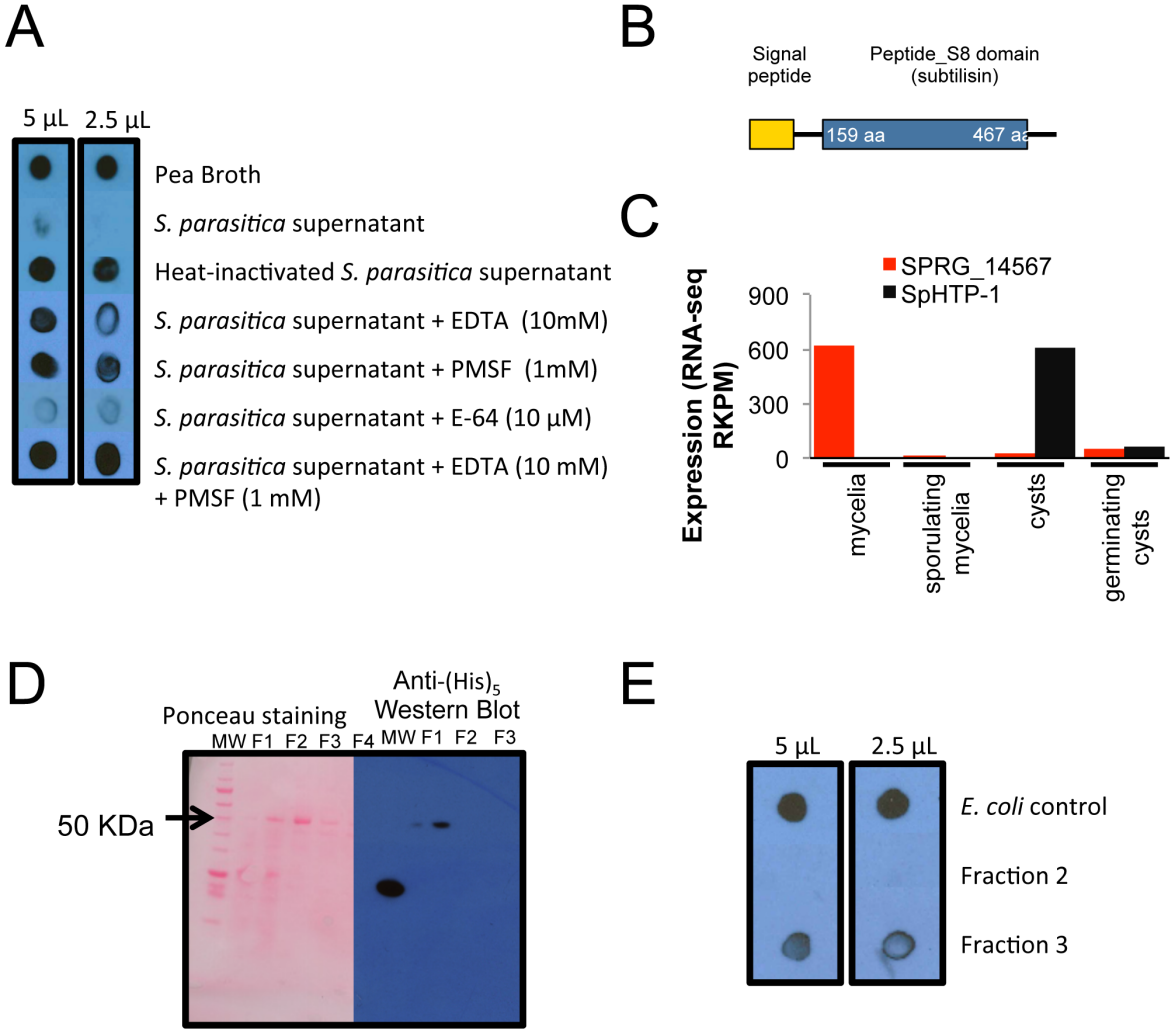
Rainbow trout IgM proteolysis by *S. parasitica* secreted proteases. (**A**) 7-day old culture filtrates were capable of degrading rainbow trout IgM after an overnight incubation at 10°C. (**B**) Schematic drawing of the domains present in the protease SPRG_14567 (**C**) Expression pattern of SPRG_14567 in different life stages. The RKPM of RNA-seq data is plotted, and the previously identified effector SpHTP-1 is plotted to show contrasting expression patterns. (**D**) The recombinant subtilisin-like protease SPRG_14567 was partially purified through tandem ion exchange (SO_3_^−^) and nickel affinity columns (Fractions 1 to 4) following detection in a Western blot using anti-(His)_5_ HRP antibody. (**E**) Fractions 2, 3 and soluble proteins from untransformed *E. coli* were tested for IgM-degrading activity with only the fraction containing the recombinant SPRG_14567 exhibiting proteolysis.

We investigated whether culture filtrates containing secreted proteins from *S. parasitica* could degrade trout immunoglobulin M (IgM) as previously found for bacterial fish pathogens [50]. Although no effect was observed when supernatants of two-day old cultures were incubated with an IgM enriched fraction (data not shown), the 7-day post-inoculation supernatant degraded the IgM protein fraction within several hours (Figure 4A). No degradation of trout IgM was detected when heat-treated 7-day post-inoculation supernatant was used. The protease inhibitors EDTA (a metalloproteinase inhibitor) and PMSF (serine protease inhibitor) showed partial inhibition of the IgM-degrading activity while E-64 (a cysteine protease inhibitor) did not show any inhibition. The combination of EDTA and PMSF prevented IgM degradation and the detection was similar to the pea broth control.

These results suggest that secreted proteases from *S. parasitica* could degrade fish IgM and that metalloproteinases and serine proteases may be the classes involved in this process. To further characterize the IgM-degrading properties of *S. parasitica* proteases, a serine protease (SPRG_14567) was selected from the *S. parasitica* genome based on our observation that this protease possesses a secretory signal peptide (Figure 4B) and is highly expressed (RNA-Seq data) (Figure 4C). Interestingly, SPRG_14567 showed strong degrading activity towards trout IgM while no activity was detected when control *E. coli* soluble proteins were used (Figure 4B and 4E). This indicates that this serine protease is capable of degrading fish IgM and could be a virulence factor that combats the activity of fish immunoglobulins against *Saprolegnia*.

### Candidate effector proteins

To establish a successful infection, pathogens often deliver effector proteins and toxins into the host cytoplasm to manipulate host immunity [7], [24], [51]. Analogous to bacterial Type II secreted toxins that enter host cells via lipid-receptor-mediated endocytosis, plant pathogenic oomycetes utilize a host-targeting domain to deliver effectors into plant cells. Hundreds of effectors carrying the host targeting motifs of RXLR and LFLAK have been identified in plant pathogenic oomycetes [11], [12], [22]. However, these large families of Crinkler and RXLR effectors appear to be absent in *S. parasitica* (**Table 2**). Using sensitive BLAST and HMM searches based on the RXLR domain and C-terminal domains of effectors, no plant pathogen-like RXLR effectors could be detected in the genome. Bioinformatic searches with the *de novo* motif-finding program MEME did not identify other putative host-targeting motifs. Despite the absence of large RXLR effector families, *S. parasitica* does have a small family of host targeting proteins related to SpHtp1, which do contain an N-terminal RXLR sequence. Interestingly one of these proteins was shown to translocate into fish cells [28] and entry required the N-terminal leader sequence [30]. The lack of sequence similarity between any part of SpHtp1 and RXLR-proteins from plant pathogen oomycetes, except for the three proximally located amino acid residues, suggests that the presence of the RXLR-sequence in SpHtp1 is currently unclear. No significant matches were found to Crinkler effectors, suggesting they are absent from *S. parasitica*. Using search criteria that do not rely on sequence homology, such as induction of expression during the pre-infection stage, presence of secretion signals, and signatures of fast evolution, several candidates (Table S10) for host-targeting proteins (including SpHtp1) were identified in *S. parasitica*. None of these candidates have homology to known proteins, and their functions are currently unknown.

The commonalities between animal and plant pathogenic oomycetes are highlighted by their shared PAMPs. Proteinaceous PAMPs found in plant pathogenic oomycetes, namely CBM1 [37], elicitins, and Cys-rich-family-3 proteins [14] are found in *S. parasitica* (Figure S7A). Elicitins are extracellular lipid transfer proteins that elicit defense responses in some species of plants, especially *Nicotiana* [52]. There are 29 elicitin-like proteins in *S. parasitica*. Phylogenetic reconstruction shows that the majority of the *S. parasitica* elicitins form three lineage-specific clades, distant from the canonical elicitin group [53] (Figure S7B). We also detected six YxSL[RK] containing secreted proteins, previously identified as candidate effectors [14], that also show sequence divergence from known members of this family. It is an intriguing question whether animal innate immune systems can detect these potential PAMPs, as does the plant innate immune system. For example it seems unlikely that CBM1 can act as immune elicitor for animal cells since binding to the plant cell wall cellulose is required to induce immune responses in plants [37].

### Elevated nucleotide substitution rate of pathogenesis associated gene families

To study the polymorphism and evolutionary rate of *S. parasitica* genes, we sequenced a related strain, VI-02736, which was isolated from an infected Atlantic salmon, for comparison. More than of 90% of the CBS223.65 reference sequence was covered by VI-02736 reads, allowing us to identify 1,467,567 SNPs between the two strains, giving an average SNP rate of 3.3% (Text S1, Table S11 and Figure S8A and S8B). We have also determined that LOH is unique to the CBS strain, as it is apparently absent in the sequenced VI-02736 strain (Text S1, Figure S8C and S8D).

The Ka/Ks ratio (Ka - number of non-synonymous substitutions per non-synonymous site, Ks - number of synonymous substitutions per synonymous site) was calculated for the annotated *S. parasitica* gene set. The set of 3518 oomycete core ortholog genes gives a median Ka/Ks ratio of 0.05, a rate comparable to previously published Ka/Ks rate of the conserved gene dense region of *Phytophthora* sibling species and related strains [54]. Non-parametric Z tests between the core ortholog group and a particular gene family (listed in Table 2) were performed to determine which family shows elevated substitution rates. Among the pathogenesis-related gene families, we identified four groups, namely elicitins, disintegrins, host targeting proteins and haemolysins, that have significantly elevated median Ka/Ks ratios as compared to the core-ortholog groups (Figure S9A). Between the two *S. parasitica* strains, the highest Ka/Ks ratio was observed in the haemolysin E family, a group of toxin-like genes that were possibly horizontally acquired from bacteria (see below). Interestingly, similar patterns of elevated Ka/Ks ratios were also found between haplotypes of the reference strain (Figure S9B). Three families, the disintegrins, elicitins and haemolysins, show significant elevations in Ka/Ks as compared to the core ortholog group (Figure S9B).

### Potential role of horizontal gene transfer in development of pathogenicity

Horizontal gene transfer events from bacteria and archaea appear to have contributed to some of the novel biosynthetic pathways found in the oomycetes [47]. By utilizing codon usage and protein domain classification (see Methods), at least 100 genes could be identified with a potential phylogenetic origin outside the super-kingdom of Chromalveolates by using the program Alien_hunter [55] and by interrogating Pfam search results. Among these, we further identified around 40 genes belonging to 5 families that could potentially be associated with pathogenesis (Table 3). Many of these genes appear to have been acquired from bacterial species, in particular from Proteobacteria. Some potential acquisitions may have occurred relatively recently as they only occur in *Saprolegnia* and seem to have nucleotide compositions predictive of foreign acquisition (Alien_hunter score >50). A caveat for our analysis is that *S. parasitica* is the only available genome so far outside of the plant pathogenic oomycetes. There are a great variety of basal oomycetes [56], [57] that do not have genome sequence information and have not been investigated. Therefore, it could be that these HGT events could have occurred in some ancestral oomycetes .

**Table 3:** Predicted horizontally transferred genes that may be associated with pathogenesis in *Saprolegnia parasitica*.

Pfam function	Functional Description	Possible Phylogenetic origin	Number Genes in the family	Representative gene ID	Subcellular Localization^a^	HGT time Estimate^b^
**Disintegrin**	Disintegrin	proteobacteria	16	SPRG_14051	secreted	recent
**Laminin like**	Associated with cell surface	-	1	SPRG_08424	secreted	recent
**CHAP**	CHAP domain	-	7	SPRG_15528	secreted	
**Endonuclease**	DNA/RNA non-specific endonuclease	bacteria	6	SPRG_08128	secreted	
**HylE**	Haemolysin E	enterobacteria	9	SPRG_04818	membrane	recent

a Subcellular localization is predicted by the N-terminal signal peptide, mitochondrial targeting motif and transmembrane domains.

b The time of horizontal gene transfer is estimated by the presence in other oomycetes and coding potential of a given gene. ‘a recently acquired gene’ refers to a gene occurring only in *Saprolegnia* and having an uncharacteristic coding potential.

Several groups of extracellular enzymes were potentially acquired from bacteria; for example, the CHAP (cysteine, histidine-dependent amidohydrolases/peptidases) family and a family of secreted nucleases (Table 3). The presence of the CHAP family (pfam hit *E* value<1e-5) is unexpected in *S. parasitica*, because it is commonly associated with bacterial physiology and metabolism [58]. These genes have undergone repeated duplications in *S. parasitica*, resulting in an expansion of gene numbers. Another potential bacterial acquisition is a family of toxin-like proteins similar to haemolysin (HlyE), a pore-forming toxin from enterobacteria such as *Salmonella* [59]. These genes have also undergone recent duplication resulting in nine copies in *S. parasitica*. Two members (SPRG_03140, SPRG_20514) of the HlyE family are expressed during infection.

Another distinctive feature of the *S. parasitica* secretome is the presence of animal-like surface proteins. The phylogenetic affinities of the two groups, distinguished by gal_lectin-like and disintegrin-like domains, suggest a possible origin via HGT, but from different sources. Gal_lectin refers to the D-galactoside binding lectin initially purified from the eggs of sea urchin [59], [60], [61], representing a group of lectins that occurs widely on fish eggs and skins [62]. Phylogenetic analysis shows that the closest homologs are animal gal_lectins (Figure 3C). The *S. parasitica* gal_lectin genes are among the most highly induced and highly expressed genes in pre-infection and infection stages, suggesting that gal_lectin may facilitate adhesion and invasion of fish cells. The gal_lectin genes contain an unusually large number of introns (7 introns), as do mammalian and fish gal_lectin genes, further suggesting a common origin. The *S. parasitica* genes have a codon usage similar to core orthologs, in contrast to other candidate HGT genes, suggesting they have been in the *S. parasitica* genome long enough to be largely assimilated. The *S. parasitica* disintegrin genes were potentially acquired from bacteria (Figure 3C, Table 3) and have since expanded. Disintegrins were initially identified as proteins preventing blood clotting in viper venoms [63]. In animals, disintegrins inhibit aggregation of the platelets by binding to the integrin/glycoprotein IIb-IIIa receptor. The crucial amino acid motif CRxxxxxCDxxExC, mediating ligand binding, is conserved in the *S. parasitica* disintegrins. All 16 disintegrin genes in *S. parasitica* are expressed in the pre-infection stages (see below), with several members belonging to the top 1% most highly expressed genes, suggesting that they may play a role interacting with animal hosts. However, we have not been able to experimentally demonstrate the role of disintegrins in pathogenesis so far (Text S1 and Figure S10).

### Tissue-specific and host-induced gene expression

*S. parasitica* has, like most other oomycetes, clearly defined life stages, including motile zoospores that are able to swim, encyst and germinate upon attachment to its host tissue. We performed strand-specific Illumina RNA-Seq analysis of 4 developmental stages (mycelium, sporulating mycelium, cysts, and germinating cysts [3–5 hours]) of *S. parasitica*, as well as a time course analysis (0, 8 and 24 hours) of a rainbow trout fibroblast cell-line (RTG-2) challenged with cysts of *S. parasitica*. RNA-Seq reads were mapped to the *S. parasitica* genome and gene annotations, and transcript abundance values were quantified as RPKM (reads per kilobase transcript length per million reads mapped).

**Figure 5:**
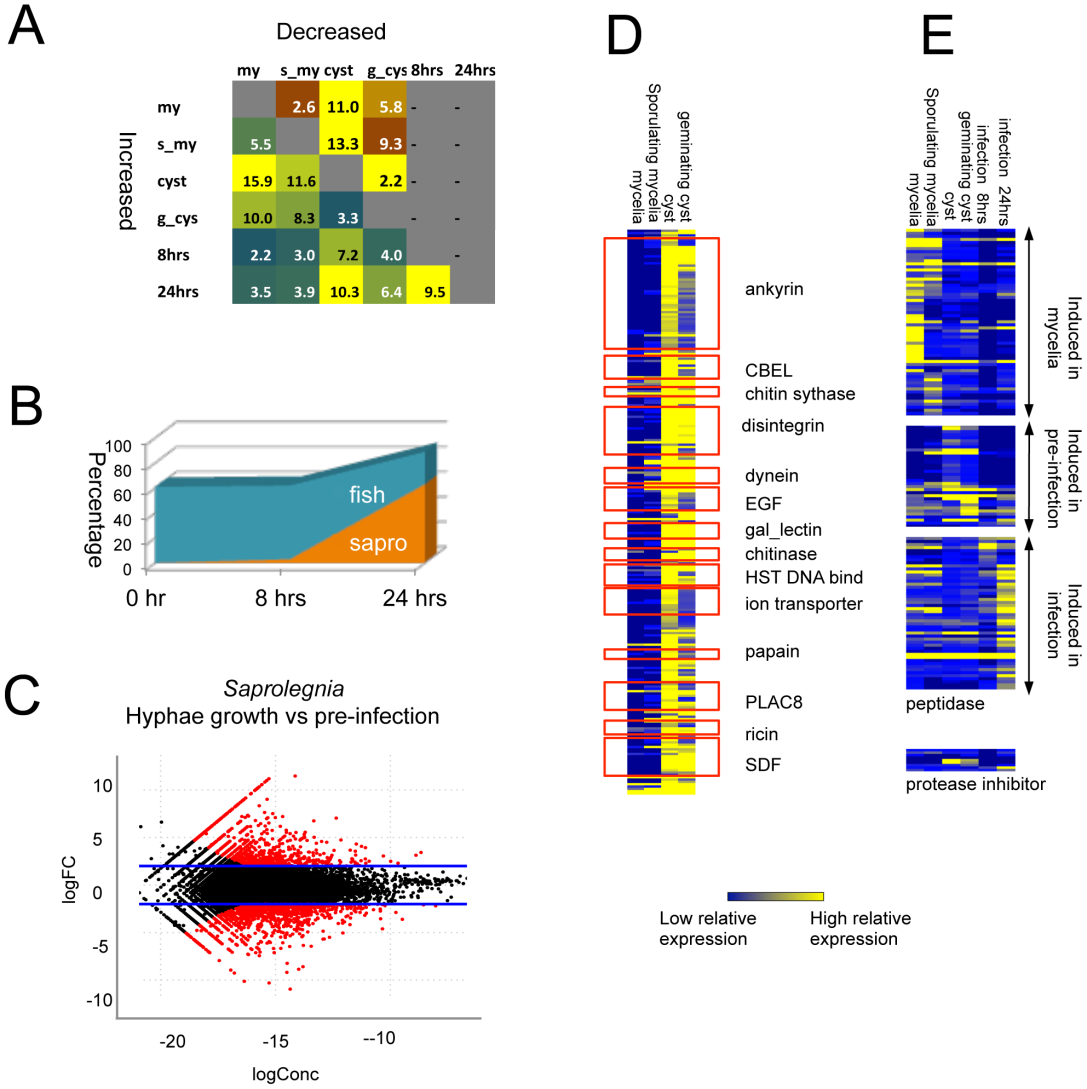
Differentially expressed genes detected by RNA-Seq. *(A)* Percentage of differentially expressed genes in pair-wise comparisons of tissue types. Genes with 4 fold RPKM (reads per kilobase per million) differences were considered to be differentially expressed (negative binomial exact test p<0.001, p value adjusted with Benjamini & Hochberg correction, Table S12) *(B)* Gene families showing differential expression between vegetative tissue (mycelia and sporulating mycelia) and pre-infection tissues (cysts and germinating cysts). CBEL:fungal Cellulose Binding Domain Like protein, EGF:(Epidermal Growth Factor, gal_lectin: Galactose binding Lectin domain, HST: Heat shock factors, PLAC8: Placenta-specific gene 8 protein, SDF: Sodium Dicarboxylate symporter Family (Table S13). *(C)* Growth phase specific expression of peptidases and protease inhibitors (Table S14). *(D)* Relative abundance of *S. parasitica* and fish transcripts during interaction. *(E)*. *S. parasitica* transcript distribution in pre-infection versus vegetative tissue. logFC = log_2_(pre-infection/vegetative/pre-infection); logConc = the log2 of average reads counts per million of each gene in the two tissue types,). Red dots indicate significant differences (p<0.001; negative binomial test).

Large numbers of genes were differentially expressed in the various life stages and conditions. Cyst and germinating-cyst stages showed similar expression profiles (linear correlation R^2^ = 0.93, p<0.001), with less than 6% of all the genes showing >4-fold expression differences (p<0.001; Figure 5A). In contrast, the other developmental life stages and infection time course showed somewhat larger differences (R^2^ of 0.84–0.88), with up to 27% of genes differentially expressed between cysts and mycelia (> = 4-fold difference; p< = 0.001). The time course experiment shows the relative abundance of the host and pathogen transcripts changing as infection progressed (Figure 5B). At the 0 hour and 8 hours time points, very few pathogen transcripts were detected (less than 1% and 3%, respectively) whereas at 24 hours, 63% of the transcripts were derived from the pathogen. At 24 hours, the transcript profile of the pathogen closely resembled that of *in vitro*-grown mycelia, with only 3.5% of all genes differentially induced between the two stages (>4 fold differences, p<0.001). Compared to the cysts used as inoculum, 7.2% of genes were induced after 8 hours of infection and 10% of genes were induced by 24 hours (>4 fold differences, p<0.001) (Figure S11A). We have defined the stage prior to host tissue colonization (germinating cyst) as the pre-infection stage. In plant pathogenic oomycetes, germinating cysts express many pathogenesis-associated genes [12], [64], [65], [66], [67]. The previously characterized *S. parasitica* gene encoding the host targeting protein SpHtp1 [28] is induced more than 100-fold in the pre-infection stage as compared to other stages. At the pre-infection stage, 10% of all genes were induced as compared to the mycelial stage (>4 fold differences, p<0.001) (Figure 5C; Figure S11B; Table S12). The profile of the germinating cysts - resembled that of the 8 hours fish cell infection (R^2^ = 0.88, p<0.001), with only 4% of genes showing induction (>4-fold differences, p<0.001).

For genes that encode proteins specific to or expanded in *S. parasitica* as compared to other oomycetes, around 25% were induced in germinating cysts compared to mycelia (>4-fold differences, p<0.001) (Figure S11C, Table S13). A total of 14 protein families showed both lineage-specific domain expansion and up-regulation in germinating cysts (Figure 5D). The largest groups are proteins carrying ankyrin domains or lectin domains, which suggests the importance of protein-protein and protein-carbohydrate interactions in the initial stages of host colonization. Another group of proteins belonging to this category are transporters such as ion transporters and sodium symporters, suggesting that metabolic exchange processes are active during the establishment of early infection.

The RNA-Seq data also suggest that the very large *S. parasitica* kinome plays an important role during the infection process. Around 10% of all kinase genes, spanning all major kinase classification groups, showed 4-fold or more induction in germinating cysts compared to mycelia (>4-fold differences, p<0.001) (Figure S11C). Many of the numerous *S. parasitica* proteases were expressed in a specific life stage or distinct point during the infection process (Figure 5D/E, Table S14). One group of peptidases (6%) was expressed in germinating cysts. These include subtilase proteins (SPRG_15005) that carry ricin lectin domains and are highly expressed in germinating cysts. A large group of peptidases (19%) were induced during the interaction with fish cells. There are 7 protease-inhibitors encoded in the genome; and they also showed patterns of differential expression (Figure 5C). The Kazal peptidase inhibitor (SPRG_09563) was most highly expressed in cysts and germinating cysts.

To confirm the RNA-seq data, we have also performed qPCR for a set of disintegrin genes, showing expression in cysts and germinating cysts, but no detection in mycelium and sporulating mycelium as seen for the RNA-seq data (Figures S11 C,D).

Taken together, the RNA-seq data reveal that members of the kinases, proteases, disintegrins and gal_lectins show upregulated expression in the pre-infection stage (Table S13 and S14), which warrants using these candidate genes for future pathogenesis related studies.

## Discussion

The genome sequence of *S. parasitica*, together with transcriptome and polymorphism data, reveals a predicted core proteome very similar to those of plant pathogenic oomycetes, but an adapted proteome strongly aligned to its animal-pathogenic lifestyle.

From the genome sequence and the RNA-Seq data we could identify several groups of proteins predicted to be secreted at the pre-infection stages that might facilitate early interactions with the hosts of *S. parasitica*. Several of these proteins seem to be unique to *S. parasitica* and may have evolved to interact specifically with fish cells. They are predicted to be targeted to the extracellular environment or incorporated into the exterior surface of the pathogen. Several of the proteins contain CBM domains (fungal cellulose binding domain), ricin-like domains, Notch-like domains and/or various peptidase domains. Most putative early interaction proteins have potential roles in pathogenesis, such as animal-cell-surface-like proteins (disintegrin, gal_lectin) and haemolysin E toxin-like proteins. Lectins could help cysts to bind to host skin. Since the lectins are highly expressed during the initial and later infection stages, we hypothesize that they play an important role in cell-cell contact throughout the interaction. This would also suggest that an intimate contact with the host cell is required for pathogenesis, suppression of host defence processes, and/or nutrient uptake. Following attachment, the pathogen engages a large arsenal of potential virulence factors in the form of proteases to attack the host tissue. Interestingly, *S. parasitica* has one of the largest numbers of proteases found in any organism. At least one protease (SPRG_14567) was found to be able to degrade IgM, suggesting an active role in suppressing initial immune responses, as fish IgM's are able to bind to infection related antigens, even in the absence of prior immunization.

In comparison to plant pathogenic oomycetes, *S. parasitica* has no canonical RXLR or Crinkler effector genes, nor NLP toxin genes in its genome. Nevertheless, one small protein family has been found for which one member was shown to translocate inside trout cells [2], [30], which suggests that the interaction of *S. parasitica* with its hosts is more subtle than a simple necrotrophic interaction based on secretion of toxins and protein degradation. In fact, we speculate that the initial stages of the interaction may involve a more ‘biotrophic’ approach by the pathogen, whereby the immune response of the host is avoided or even suppressed during initial colonization via, for example, proteases or effector proteins. Following this biotrophic stage, the host tissue is bombarded with proteases, lipases, and lysing enzymes. If *S. parasitica* was a plant pathogen, it would thus have been classified as a ‘hemi-biotroph’ and not a saprotroph as its name would suggest. Further experiments are required to demonstrate that this is indeed the case.

Pathogenicity towards animals has evolved independently in both the fungi and oomycetes. It has also evolved at least three additional times within the kingdom Stramenopila: within the Pythiales (Figure 1A), within the genus *Aphanomyces* [6] and in the non-oomycete *Blastocystis*. Analyses of the genomes or sequenced ESTs of these other pathogens reveals some interesting parallels. For example, based on only a small set of ESTs of the oomycete human pathogen *Py. insidiosum* [29], an identified expressed lectin CBM-encoded transcript has been implicated in the process of pathogenesis. *Blastocystis hominis* is a Stramenopile human pathogen with a very small genome of 19 Mb encoding only 6020 genes, and is phylogenetically very divergent from oomycetes [4]. Despite the large evolutionary distance, the genome of *Blastocystis* shows reduction of nitrogen and sulfite metabolic pathways, similar to what is seen in obligate plant pathogens. In addition, similar to the acquired lectins in *S. parasitica*, *Blastocystis* has also horizontally transferred genes with animal like features, such as genes encoding Ig domains [4]. The fungal pathogen *Batrachochytrium dendrobatidis* that causes global amphibian decline, inhabits an aquatic environment like *S. parasitica*, and also causes diseases on animal skin and tissues. Analysis of its genome revealed patterns of expansion of protease families [68]. Although the particular families are different, *B. dendrobatidis* possesses several families of lineage expanded proteases such as metallopeptidase (M36).

The extensive LOH observed in *S. parasitica*, covering approximately one-third of the genome and also the high rate of polymorphism (2.6%) in the remainder of the genome are similar to recent observations in the genome of the oomycete plant pathogen *Phytophthora capsici*, where LOH was found to be associated with changes in mating type and pathogenicity [13]. We speculate that, as in *P. capsici*, LOH may provide a mechanism in *S. parasitica* for rapidly expressing diversity within a population, fixing alleles, and enabling rapid adaptation to its environment.

**Figure 6:**
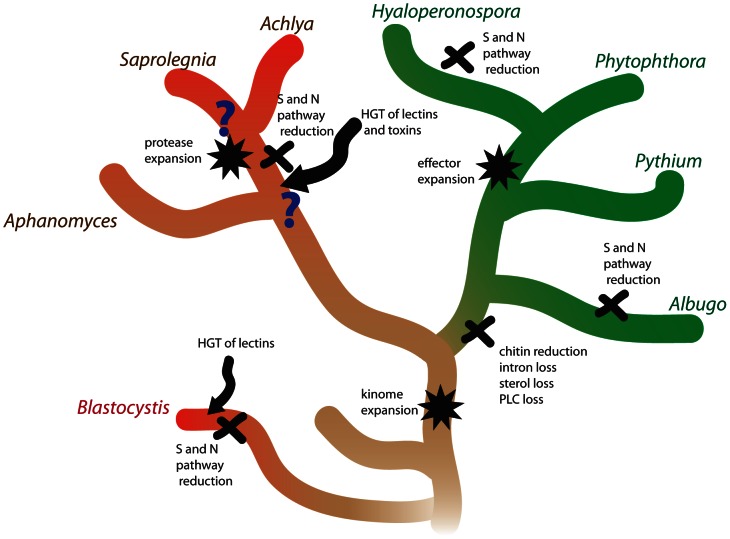
Molecular genetic events associated with the evolution of animal and plant pathogenesis in oomycetes. The lineages of animal pathogens are colored red and the lineages of plant pathogens are colored green. The basal lineage is colored brown. S and N pathways refer to sulfite and nitrite assimilation, respectively.

The evolution of plant and animal pathogenesis in oomycetes has been associated with several major molecular events (Figure 6). Since both *Phytophthora* and *Saprolegnia* have large kinomes, the expansion of kinases is likely a relatively early event in oomycete evolution. The comparison of the animal pathogen *S. parasitica* with plant pathogens with different lifestyles has shown that surviving on ammonium rich tissue has led to a reduction of metabolic pathways independently in *S. parasitica* and obligate oomycete plant pathogens. Similar reductions have also occurred in obligate fungal pathogens [69] and in the distantly related stramenopile human pathogen *Blastocystis* [4]. The evolution of plant pathogenicity has been associated with a series of reduction events such as intron loss, chitin loss and sterol loss. Some of these losses may be due to evasion of plant immunity; for example chitin can act as a PAMP to trigger plant defense responses. The evolution of plant pathogens has been accompanied by expansions of large repertoires of effectors, which have been shown to modulate plant host physiology. In contrast, the development of animal pathogenesis has been facilitated by expansion of proteases and horizontally acquired lectins and toxins.

## Methods

### Strain selection and genomic DNA isolation

*Saprolegnia parasitica* isolate CBS223.65 was isolated from young pike (*Esox lucius*) in 1965 and obtained from Centraal Bureau voor Schimmelcultures (CBS), the Netherlands. *Saprolegnia parasitica* isolate VI-02736 (N12) was obtained from parr of Atlantic salmon in Scotland in 2002 (Lochailort) [70] and kindly provided by Dr. Ida Skaar (Norwegian Veterinary Institute). Both isolates were maintained on potato dextrose agar (Fluka). For genomic DNA isolation, *S. parasitica* was grown for three days at 24°C in pea broth (125 g L^−1^ frozen peas, autoclaved, filtered through cheese cloth, volume adjusted to 1 L and autoclaved again). Genomic DNA extraction was adapted from Haas *et al* [12]. Fresh mycelia (∼1 g) were ground to a fine powder under liquid nitrogen, mixed with 10 mL of extraction buffer (0.2 M TrisHCl pH 8.5, 0.25 M NaCl, 25 mM EDTA, 0.5% SDS), 7 mL Tris-equilibrated phenol and 3 mL of chloroform∶isoamyl alcohol (24∶1), incubated at room temperature for 1 hr and centrifuged at 6,000 g for 30 m. The aqueous phase was extracted with equal volume of chloroform∶isoamyl alcohol (24∶1) and centrifuged at 10,000 g for 15 min. 50 µl of 10 mg/ml RNase A was added to the aqueous phase and incubated for 30 min at 37°C. Isopropanol (0.6 volumes) was added, mixed gently, and then the DNA was precipitated on ice for 30 min. DNA was collected by centrifugation at 10,000 g for 20 min, washed with 70% ethanol, dried and resuspended in DNase/RNase-free water. DNA was checked for quality and RNA contamination by gel electrophoresis using 0.8% agarose.

### Collection of samples and RNA isolation

The life stages of *S. parasitica* were harvested as described by van West *et al.* [28]. Zoospores and cysts were collected by pouring the culture filtrate through a 40–70 µm cell strainer and concentrated by centrifugation for 5 min at 1500 g. RNA was isolated from mycelia and sporulating mycelia using the RNeasy kit (Invitrogen) according to the manufacturer's protocol. RNA from zoospores, cysts and germinating cysts was resuspended in TRIzol (Invitrogen) and aliquoted as 1 ml portions in 2-ml screw-cap tubes containing 10–35 glass 1 mm diameter beads (BioSpec) and immediately frozen in liquid nitrogen. Frozen cells were processed with a FastPrep machine (ThermoSavant) and shaken several times at a speed of 5.0 for 45 sec until defrosted and homogenized. TRIzol RNA isolation was then performed according to manufacturer's recommendations (Invitrogen).

Host-induced gene expression was measured in rainbow trout (*Oncorhynchus mykiss*) gonadal tissue continuous cell line RTG-2, obtained from the American Type Culture Collection (ATCC CCL-55) [71]. RTG-2 cells were grown to a monolayer at 24°C (no CO_2_) and washed twice with Hank's Balanced Salt Solution (HBSS). Cells were harvested in approximately 5 ml of HBSS and *S. parasitica* cysts were added (5×10^4^ cysts per 75 cm flask) and the mixture was then incubated at room temperature for 15 min. At this time, the time 0 hr (T0) stage sample was collected, by gently pouring off medium (and also loose/non-attached cysts) and then adding TRIzol reagent for RNA extraction. Other samples were incubated for 8 hr (T8) or 24 hr (T24) at 24°C (no CO_2_) in Leibovitz (L-15) growth medium (Gibco) supplemented with 10% foetal calf serum (Biosera), 200 U ml^−1^ penicillin and 200 µg ml^−1^ streptomycin (Fisher). Cells were harvested for RNA extraction by pouring off medium and adding TRIzol. Cells were loosened using a cell scraper (Fisher) and the suspension was aliquoted as 1 ml portions into 2-ml screw-cap tubes containing 10–35 glass 1 mm diameter beads (Biospec) and immediately frozen in liquid nitrogen. Frozen cells were processed using a FastPrep machine and shaken several times at a speed of 5.0 for 45 sec until defrosted and homogenized. TRIzol RNA isolation was then performed according to manufacturer's recommendations.

Infected and uninfected fish tissue was collected from a trout (*O. mykiss*, caught in a commercial Scottish hatchery) that showed lesions caused by *S. parasitica* infection. Tissue was collected from the lesion (body) and from an infected anal fin. As an uninfected control, similar tissue not showing symptoms of infection was collected from the same fish. Tissue was ground up in liquid N_2_ and RNA was isolated using the RNeasy kit (QIAGEN) according to manufacturer's recommendations.

Infected and uninfected Atlantic salmon (*Salmo salar*) egg samples were collected from a commercial Scottish hatchery. Five eggs per sample were ruptured using a needle and placed into 5 ml of TRIzol and RNA was isolated according to manufacturer's recommendations with an additional phenol-chloroform extraction step. Multiple sample batches were pooled to obtain sufficient material for RNA-Seq library construction.

All RNA samples were resuspended in DNase/RNase-free water and treated with Turbo DNA-free DNase (Ambion) according to manufacturer's recommendations. RNA was checked for quantity and purity using a Nanodrop spectrophotometer (Thermo Scientific) and 1% agarose gel electrophoresis. Samples were stored at −80°C and were not defrosted until use.

### 454 and Sanger sequencing for genome assembly

454 fragment and 3 kb jumping whole genome shotgun libraries were generated for isolate CBS 223.65 as previously described [72]. Libraries were sequenced with ∼400 base single end reads (Titanium chemistry) using a 454 GS FLX sequencer following the manufacturer's recommendations (454 Life Sciences/Roche). Approximately a total of 22-fold sequence coverage was generated from fragment and 3 kb jumping libraries combined. A 40 kb insert Fosmid whole genome shotgun library from isolate CBS 223.65 was generated using the EpiFOS fosmid cloning system following manufacturer's recommendations (Epicentre). The Fosmid library was end-sequenced with ∼700 bp reads using Sanger technology to approximately 0.3-fold coverage using a 3730xl DNA analyzer following manufacturer's recommendations (Applied Biosystems/Life Technologies).

### Illumina sequencing for variant detection and RNA-Seq

For variant calling, Illumina whole genome shotgun fragment libraries were generated for isolates CBS 223.65 and VI-02736 as previously described [73] and sequenced with 76 base paired-end reads to a minimum of 70-fold sequence coverage using an Illumina Genome Analyzer II (Illumina) following the manufacturer's recommendations. Illumina strand-specific dUTP RNA-Seq libraries were generated for all RNA samples as previously described [74] with the following modifications. The mRNA was processed using the Dynabeads mRNA purification kit (Invitrogen) and incubated with RNA fragmentation buffer (Affymetrix) at 80°C for 1.5 to 3.5 minutes depending sample quality. Indexed adaptors for Illumina sequencing were ligated onto end-repaired, A-tailed cDNA fragments by incubation with 4,000 units of T4 DNA ligase (New England Biolabs) in a 20 ml reaction overnight at 16°C. 16 to 21 cycles of PCR were used to amplify sequencing libraries. Libraries were purified using 1–3 rounds of AMPure beads (Beckman Coulter Genomics) following manufacturer's recommendations. Libraries were sequenced with 76 base paired-end reads using an Illumina Genome Analyzer II following manufacturer's recommendations (Illumina) generating a total of 124 million reads.

### *Saprolegnia parasitica* CBS 223.65 *de novo* genome assembly

Sanger Fosmid paired-end reads were quality trimmed using the ARACHNE ‘Assemblez’ module (http://www.broadinstitute.org/crd/wiki/index.php/Assemblez). Read headers were amended to contain template and pairing information. Sanger, 454 fragment and 3 kb jumping reads were assembled using 454's Newbler assembler version 04292009 (454 Life Sciences/Roche). The resulting assembly was further processed using the ARACHNE ‘HybridAssemble’ module (http://www.broadinstitute.org/crd/wiki/index.php/HybridAssemble) using the 454 assembly, Sanger and 454 read data as input with option ‘RecycleBadContigs’ turned off allowing for extra copies of repeat sequences to be assembled. The Arachne ‘AddReadsAsContigs’ module was run to allow assembly of additional repetitive sequence. The assembly was screened for known sequencing vector sequences using BLAST and contigs with hits to known sequence vectors were removed. The assembly was further post-processed by removing contigs and scaffolds less than 200 bp and 2 kb in length respectively.

### SNP calling

Illumina shotgun sequence data were used to identify polymorphisms in two *S. parasitica* strains (CBS 223.65 and VI-02736). Illumina paired-end fragment reads from strains CBS 223.65 and VI-02736 were independently aligned to the *S. parasitica* CBS 223.65 reference assembly using the BWA aligner [75] using default settings. Read alignments were sorted by scaffold and position along the reference assembly. SNP calling was performed using the GATK Unified Genotyper module [76]. Variant Call Format (VCF) files containing SNP calls were filters for low quality using parameters: AB>0.75 && DP>40 ∥ DP>500 ∥ MQ0>40 ∥ SB>−0.10. SNP calls for CBS 223.65 and VI-02736 can be retrieved from the Broad Institute *Saprolegnia parasitica* genome database website (http://www.broadinstitute.org/annotation/genome/Saprolegnia_parasitica/MultiDownloads.html).

### Loss of heterozygosity and haplotype region identification

SNP calls and depth of read coverage information were parsed from the VCF file described above and analyzed in non-overlapping 5 kb windows (Figure S1). Using this information genome segments were partitioned into three groups: separated haplotypes (coverage depth ranging 20–55-fold and SNP rate <1%), diploid homozygous involving LOH (coverage depth ranging 56–90-fold and SNP rate <1%), and diploid heterozygous (coverage depth ranging 40–90-fold and SNP rate > = 1%). Coverage of separated haplotype regions peaks at ∼40-fold and the regions are mostly devoid of SNPs. The region corresponding to the diploid consensus exhibits ∼60-fold coverage and nearly a 3% SNP rate. The peaks in Figure S1B corresponding to the separated haplotype and consensus diploid regions are connected by a small ridge, which correspond to windows spanning boundaries between the different kinds of regions. The coverage for the diploid consensus regions is not exactly double as compared to the predicted separately assembled haplotype regions, and is less than the diploid homozygous regions; most likely this results from the relative difficulty of aligning short Illumina reads to diploid consensus sequences in the context of the high polymorphism rate observed.

Individual genes located in haplotype contigs were assigned as likely allelic pairs based on SNP rate, depth of coverage, and taking into consideration best reciprocal blast matches and synteny between separately assembled haplotype contigs.

### Genome annotation

Gene finding used both evidence-based (including EST, RNA-Seq and homology data) and *ab initio* methods. Gene-finding algorithms FGenesH, GeneID and GeneMark were trained for *S. parasitica* using existing gene and EST datasets. Then a statistical sampling of gene calls as well as genes of interest were manually curated, and the results were used to validate gene calls and fine-tune the gene caller. RNA-Seq data was incorporated into gene structure annotations using PASA [77] as described in Rhind *et al*. [78]. Subsequently, the annotated total gene set was subjected to Pfam domain analysis, OrthoMCL clustering analysis and KEGG metabolic pathway analysis.

### Expression analysis with RNA-Seq

Illumina RNA-Seq data was processed as follows. Sequencing adaptors were identified and removed from reads by exact match to adaptor sequences. Reads were aligned to *S. parasitica* gene transcripts using Bowtie (allowing up to 2 mismatches per read, and up to 20 alignments per read). Transcript levels were calculated as FPKM (fragments per kilobase cDNA per million fragments mapped). The program EdgeR [79] was used to identify differentially expressed transcripts. Transcripts with significantly different levels (p< = 0.001 and over 4-fold difference) were identified, and p-values were adjusted for multiple testing by using the Benjamini & Hochberg [80] correction.

### Metabolism analysis

The predicted proteomes of *S. parasitica* and representative plant pathogenic oomycetes were annotated by mapping against a reference set of metabolic pathways from KEGG (Kyoto Encyclopedia of Genes and genomes) [81]. The method used, KAAS (KEGG Automated Annotation Server), utilizes bidirectional best hits to assign pathways. Subsequently, the metabolic genes and pathways were manually annotated and compared to other oomycete pathogens. Known genes involved in nitrogen and sulfur metabolism were used to search the *S. parasitica* genome by TBLASTN search; genes were considered to be candidates if a positive hit was found (E value<1e-5). For chitin metabolism analysis, genes were annotated based on Pfam homology (E value<1e-5) with the exception of chitin synthase genes, which were identified after Blastp analysis against a set of oomycete and fungal CHS. A total of twelve GH18 genes were identified, of which six are arranged in small clusters of two paralogous genes.

### Horizontal gene transfer analysis

Two different approaches were used to screen *S. parasitica* genes for candidate HGT origins. In the first approach, the genome sequence was screened with the program Alien_hunter [55]. The program utilizes an interpolated variable order motif method to determine horizontally transferred events, purely based on compositional difference between a region and the whole genome framework. Because the methodology is independent of any existing datasets, we used it to examine the *S. parasitica* genome. Genomic regions were identified as alien when the Alien_hunter score was above 50. Out of 1442 *S. parasitica* supercontigs, 206 supercontigs had distinct regions marked as alien after running Alien_hunter. Subsequently, the 1616 gene models that lay within the candidate alien regions were extracted and compared with other oomycete genomes (*P. sojae*, *P. ramorum*, *P. infestans*, *H. arabidopsidis* and *Py. ultimum*). In the second approach, the entire proteome of each oomycete was scanned for homology to Pfam-A protein families using the hmmscan algorithm from Hmmer 3.0 applied to the Hidden Markov Model dataset (Pfam-A.hmm v.24). A cut-off e-value threshold of 1e-3 was applied. From the Pfam domain analysis we obtained 307 sequences that had distinct domains not found in any of the *Phytophthora* species, and 31 of the candidates derived from the Pfam analysis overlapped with the results from Alien_hunter. We then blast-searched all 1616 genes from Alien_hunter and the 307 genes from the domain analysis against the NCBI non-redundant database (nr) to obtain the primary functions. Phylogenetic analysis using neighbor joining was then performed on the final set of genes.

### Analysis of secreted proteases

*S. parasitica* CBS 223.65 mycelium plugs were grown in pea broth for 2 or 7 days. The culture supernatants were harvested, centrifuged at 5000× g for 10 min (4°C) and the soluble fraction used as a source of secreted proteases. The ammonium sulfate precipitated fraction from rainbow trout serum [82] was used as source of fish IgM (10 mg/mL of total protein concentration). For protease activity experiments, 50 µL of the culture supernatants were incubated overnight at 10°C with 5 µL of the IgM enriched fraction. Pea broth was used as a negative control. Heat-inactivated supernatant was obtained by incubating the culture supernatants for 15 min at 95°C. The protease inhibitors EDTA (10 mM), PMSF (1 mM) and E-64 (10 µM) were used to identify classes of proteases involved in IgM degradation.

Two samples volumes (2.5 and 5 µL) were spotted onto nitrocellulose membranes, allowed to dry for 45 min, blocked with milk-PBS (5% dry-milk) and remaining IgM was detected using a monoclonal anti-trout/salmon HRP conjugated antibody (Aquatic Diagnostics). SPRG_14567 was cloned from *S. parasitica* CBS223.65 cDNA into the pET21B vector. *E. coli* Rossetta-gami B competent cells were transformed with the expression vector by heat-shock. Transformed cells were grown in modified LB media (100 mM NaHPO_4_, pH 7.4; 2 mM MgSO_4_, glucose 0.05% w/v; and NaCl 0.5% w/v). When *E. coli* cells reached an OD_600_ of 0.8, cultures were induced with IPTG (10 mM) and incubated at 200 rpm at 37°C for 12 hr. The soluble fraction was purified from French press supernatant by tandem ion exchange (on SO_3_^−^) and nickel affinity columns. Chromatography fractions were submitted tor 1D SDS-gel electrophoresis (NuPAGE Electrophoresis System, Invitrogen) and then transferred to nitrocellulose membranes (XCell II Blot module, Invitrogen). Membranes were blocked with milk-PBS-T (0.02% v/v Tween, 10% dry milk) for 20 min. Anti-(His)_5_ HRP conjugate (QIAGEN) was added to a final dilution of 1∶20,000 and incubated at room temp for 1 hr for detection of the recombinant protein. Chromatography fractions were tested for IgM degrading activity as previously described [50]. Briefly, 50 µL (5 µg/mL of total protein concentration) of the fractions were incubated overnight at 10°C with 5 µL of the IgM enriched fraction. Untransformed *E. coli* soluble proteins were used as a control. Remaining IgM was then detected in a dot-blot as described above.

### Phylogeny reconstruction and domain combination analysis

Gene models were analyzed using TMHMM and ClustalX. For phylogeny reconstruction, sequences were aligned using ClustalX and aligned sequences were subjected to phylogenetic analysis (NJ) using PAUP. The predicted proteome of *S. parasitica* was compared to that of six pathogenic oomycetes together with sixty-four other eukaryotic species covering all major groups of the eukaryotic tree of life, as previously used by Seidl *et al.* [48]. We used hmmer-3 [http://hmmer.org] and a local Pfam-A database to predict 1,798,601 domains in 862,909 proteins of which 19,896 domains in 10,887 proteins are found in *S. parasitica*. The architectures of multi-domain proteins were analyzed from the N- to the C-terminus, which identified 18,512 domain combinations consisting of two contiguous domains. Of these there were 1120 oomycete-specific domain combinations encoded by 2286 proteins. The great majority of combinations were specific to a single species, and only 58 combinations were found in more than one oomycete species. *S. parasitica* contained 338 domain combinations that are specific for oomycetes including 169 domain combinations encoded by 215 genes that are specific for this species (Table S9).

### Data deposition

*Saprolegnia parasitica* CBS 223.65: Sanger sequence data were submitted to the NCBI Trace Archive (http://www.ncbi.nlm.nih.gov/Traces/trace.cgi) and can be retrieved using query: CENTER_NAME = “BI” and CENTER_PROJECT = “G1848”. 454 and Illumina sequence data were submitted to the NCBI Short Read Archive (SRA) (http://www.ncbi.nlm.nih.gov/sra) and can be retrieved using the following accession numbers: 454 fragment reads (SRX007896, SRX007901, SRX007898, SRX007895, SRX005344, SRX007971, SRX007958, SRX005346); 454 3 kb jumping reads (SRX007902, SRX007899, SRX007937, SRX007574, SRX007903); Illumina fragment reads (SRX022535); Illumina RNA-Seq reads (BioProject 164643: mycelium SRX155934, sporulation mycelium SRX155933, cysts SRX155932, germinating cysts SRX155938, infected fish cell-line t = 0 SRX155937, infected fish cell-line t = 8 SRX155936 and infected fish cell-line t = 24 SRX155935. BioProject 167986: infected fish tissue SRX155944, uninfected fish tissue SRX155942, infected salmon eggs SRX155943, SRX155940 and uninfected salmon eggs SRX155941). Draft genome assembly sequence was submitted to GenBank (BioProject ID 36583, accession ADCG00000000).

*Saprolegnia parasitica* VI-02736 : Illumina sequence data were submitted to the SRA (BioProject 164645, accession SRX155939).

SNP calls for strains CBS 223.65 and VI-02736 can be downloaded from the Broad Institute *Saprolegnia parasitica* genome database website (http://www.broadinstitute.org/annotation/genome/Saprolegnia_parasitica/MultiDownloads.html).

## Supporting Information

Figure S1 (A) Illumina read coverage of the assembly. The coverage was examined at 100 base intervals, yielding peak coverage of approximately 50× (B) Frequency of nucleotide positions with given *k*-mer coverage. The mean *k*-mer coverage (38.4) of haplotype alleles was calculated from the first Gaussian curve (colored red). The mean *k*-mer coverage (59.7) of the single copy sequences was calculated from the second Gaussian curve (colored orange) fitted to the main peak. The single copy sequences' coverage was used to calculate the total genome size. Relative abundance from 0 to 1 of nucleotides was plotted. The fitted Gaussian curves have R^2^ >0.999 (P<1e-10). (C) Illumina read coverage and polymorphism rates averaged across non-overlapping 5 kb genomic regions. Color bar indicates numbers of 5 kb regions. The plot shows partitioning of the genome segments into three groups: separated haplotypes, diploid homozygous involving LOH, and diploid heterozygous (as defined in Text S1).
(PDF)

Figure S2 Gene content differences between *S. parasitica* and *Phytophthora*. *(A)* Number of genes orthologous between *S. parasitica* and *P. infestans*. The core proteome that is conserved among multiple *Phytophthora* species is indicated with a dark green circle. The phytopathogen core proteome derived from *P. infestans*, *P. ramorum*, *P. sojae*, *Pythium ultimum*, and *Hyaloperonospora arabidopsidis. (B)* Core proteome differences between *S. parasitica* and *Phytophthora*. Core protein sequences from *P. sojae* (green) and *S. parasitica* (orange) are ordered by their amino acid similarity to orthologous *P. infestans* proteins. Sequences with high similarity (>50%) are shown in solid, while those with less similarity (between 50% and 30%) are shown with a line. Sequences with less than 30% are not shown.
(PDF)

Figure S3 Mobile element comparison between *S. parasitica* and *Phytophthora*. *(A)* Mobile elements in *S. parasitica* and *Phytophthora*. The average copy number in *P. infestans*, *P. sojae* and *P. ramorum* is used as the copy number for *Phytophthora*. The elements are sorted based on the estimated copy number. *(B)* The *S. parasitica* element LTR-Sp1 is similar to the Copia-like family (Q572G9_PHYIN) in *Phytophthora* species. *(C)* The *S. parasitica* line element Line-Sp1 shows most homology with LINE elements found in fish and amphibian species (no other similar elements were found in other animal species). SwissProt protein species codes were used to name the sequences. The phylogenetic tree was constructed by using the neighbor joining method with 5000 replicates for bootstrap analysis.
(PDF)

Figure S4 The expanded kinome of *S. parasitica*. *(A)* The distribution of *S. parasitica* kinases compared to other organisms. The kinases are named after the Standard Kinase Classification Scheme at kinase.com. TK = tyrosine kinase; TLK = TK-like; STE = STE7,11,20 family of MAP kinases; CMGC = (CDK, MAPK, GSK3 and CLK) family; CK1 = cell (casein) kinase 1 family; CAMK = Calmodulin/Calcium modulated kinase family; AGC = Protein Kinase A, G, and C families. The unclassified kinases are indicated in black. *(B) S. parasitica* contains a large number of protein kinases that contain trans-membrane helices. Pies are scaled to the total number of kinases in each species. *(C)* Kinase genes that are induced in the germinating cyst stage in *S. parasitica* compared to mycelia. Transcripts elevated more than four-fold relative to vegetative stages are considered to be induced.
(PDF)

Figure S5 (*A*) Phylogram of PLCYc domains of *S. parasitica* PLC1 and PLCs from various organisms. For phylogenetic analysis, the PLCYc domains were determined by Smart (http://smart.embl-heidelberg.de), alignments were made and regions containing gaps were eliminated resulting in a total of 88 positions in the final dataset. The optimal tree was inferred using the Neighbor-Joining method with 5000 replicates and constructed using MEGA version 4. PLC sequences were derived from NCBI (*), JGI databases (http://genome.jgi-psf.org/,#), the Sanger Institute (http://www.genedb.org,), http://bioinformatics.psb.ugent.be,). *Arabidopsis thaliana* AtPLC1 (Q39032*), AtPLC2 (Q39033*), AtPLC3 (Q56W08*), AtPLC4 (Q944C1*), AtPLC5 (Q944C2*), AtPLC6 (UPI000034EE4D*), AtPLC7 (Q9LY51*), AtPLC8 (Q9STZ3*), AtPLC9 (Q6NMA7*); *Aureococcus anophagefferens* (Auran; 18506#); *Ciona intestinalis* (Cioin; XP_002129990*); *Cryptosporidium parvum* (Crypa; Q5CR08*); *Danio rerio* (Danre; XP_689964*); *Ectocarpus siliculosus* (Ectsi; Esi0000_0131$); *Emiliania huxleyi* (209393#); *Fragilariopsis cylindrus* (Fracy;186252#); *Homo sapiens* (as described by [9]*); *Naegleria gruberi* (Naegr; 1225#); *Paralichthys olivaceus* (Parol; ACA05829*); *Paramecium tetraurelia*, (PLC1, see [10]*); *Phaeodactylum tricornutum* (Phatr; 42683#), *Plasmodium falciparum* (Plasmo; PF10_0132@); *Salmo salar* (Salsa; NP_001167177*); *S. parasitica*: Sap-PLC (SPRG_04373#), *Thalassiosira pseudonana* (Thaps; 263246#), *Toxoplasma gondii* (Toxgo; XP_002367229*). (*B*) Gene structure of PLC genes. PLC is missing from other sequenced oomycete genomes, but present in *S. parasitica*. Multiple introns have been identified in the *S. parasitica* PLC gene.
(PDF)

Figure S6 Sterol biosynthetic pathway inferred in *S. parasitica*. *(A)* The pathway from acetyl-CoA to lanosterol. *(B)* The pathway from lanosterol to zymosterol. The red box shows CYP51 sterol demethylase, a target of azole anti-fungal chemicals. *(C)* Pathways from zymosterol to cholesterol and fucosterol.
(PDF)

Figure S7 Phylogenetic distributions of infection-related molecules. *(A)* Classes of infection-related molecules. Two groups of PAMPs, elicitin-like and cys-rich-family-3 proteins are present in both animal- and plant-pathogenic oomycetes (colored red). The gray dots indicate infrequent occurrences. *(B)* Elicitin-like proteins in *S. parasitica* and *Phytophthora*. The canonical *Phytophthora* and *Pythium* elicitins are colored green. *S. parasitica* elicitin-like proteins are divergent and form species-specific clades.
(PDF)

Figure S8 Distribution of rates of polymorphisms. (A) Summary of SNP content across 5 kb regions for Saprolegnia CBS and N12 strains. (B) Density of SNPs according to 5 kb regions of the CBS genome. The mode for the SNP rate is 2.6%. The bulge on the left side of the distribution likely corresponds to 5 kb regions of the assembly that are mosaic between haplotype and consensus diploid, as can be seen having overlap in the distribution shown in the contour plot (Figure S1C). (C) Distribution of rates of polymorphisms between strains CBS and N12. Both heterozygous and homozogous polymorphic sites were considered across 5 kb regions of the CBS genome with Illumina reads aligned from strain N12. The mode for the %SNP was computed as 3.1%. (D) Distribution of rates of polymorphisms within strain N12. Only heterozygous sites were examined in the alignments of Illumina N12 reads to the CBS strain's genome. The mode for the %SNP was computed to be 1.7%.
(PDF)

Figure S9 *(A)* Nucleotide substitution rate between *S. parasitica* strain CBS223.65 and N12. Asterisks indicate significant differences between the gene family and the core orthologs (* p<0.001; ** p<10^−5^) based on a non-parametric Z-test. *Phytophthora* data is based on the published results of Raffaele *et al*. (2010). GSR (Gene Sparse Region), GDR (Gene Dense Region). (B) Nucleotide substitution rate between the separated haplotypes of the strain of *S. parasitica* strain CBS223.65.
(PDF)

Figure S10 Predicted disintegrin SPRG_14052 does not enter fish cells *in vitro*. *(A)* Amino acid sequence of fusion protein SPRG_14052_mRFP-His_6_. The CRxxxxxCDxxExC disintegrin motif is shaded in red. The mRFP sequence is indicated in blue, the His-tag is in green. *(B)* RTG-2 cells were exposed to 3 µM of mRFP, SpHtp1 or SPRG_14052_mRFP-His_6_ and incubated for 30 min, before photography.
(PDF)

Figure S11 Stage specific gene expression detected by RNA-Seq in the total gene set. *(A)* Genes differentially expressed during fish cell interaction. *(B)* Differentially expressed genes in different life stages. *(C)* The correlation coefficients of pairwise comparisons between RNA-Seq data sets (p<0.001). *(D)* Transcript levels of a subset of disintegrin-encoding genes in various life stages of *S. parasitica* determined by RNAseq and qPCR. For RNAseq, the log2 value of RKPM of a gene is plotted. For qPCR, transcript levels are relative to the transcript levels of SpHtp1 in cysts and normalized against the reference gene SpTub-b encoding for tubulin. Error bars correspond to four biological replicaties.
(PDF)

Text S1 Supplementary information on: phospholipid modifying enzymes and signaling enzymes, sterol metabolism, disintegrin-like proteins and supplementary methods.
(DOCX)

Table S1 *Saprolegnia parasitica* genome assembly statistics.
(DOCX)

Table S2 Assembled *S. parasitica* genome partitioned into classes based on coverage and polymorphisms.
(DOCX)

Table S3 *S. parasitica* genes and SNPs percentages.
(TXT)

Table S4 Chitin biosynthesis, modification and degradation in oomycetes.
(DOCX)

Table S5 Gene encoding nitrogen and sulphur assimilation enzymes in oomycetes.
(DOCX)

Table S6 Phospholipid modifying and signaling enzymes in *Saprolegnia parasitica* and other oomycetes.
(DOCX)

Table S7 Unique and expanded domains in *Saprolegnia parasitica* proteome.
(DOCX)

Table S8 *Saprolegnia parasitica* secretome.
(XLSX)

Table S9 Protein domain combinations in *Saprolegnia parasitica*.
(XLSX)

Table S10 Candidate effectors in *Saprolegnia parasitica*.
(XLSX)

Table S11 Polymorphism statistics for *Saprolegenia* strains.
(XLSX)

Table S12 Genes that are significantly induced in cyst and germinating cyst stages as compared to the mycelial tissue by RNA-seq experiment.
(XLSX)

Table S13 Genes encoding lineage-expanded domains that are Iinduced in cysts or germinating cysts stage.
(XLSX)

Table S14 Expression of peptidase genes in *S. parasitica* by RNA-seq experiments.
(XLSX)
