# Supplementary figures and images for: Distinctive Expansion of Potential Virulence Genes in the Genome of the Oomycete Fish Pathogen Saprolegnia parasitica
Source: PLoS Genet. 2013 Jun 13;9(6):e1003272. doi: 10.1371/journal.pgen.1003272 (PMC3681718; doi:10.1371/journal.pgen.1003272)

# Supplementary Figure S1

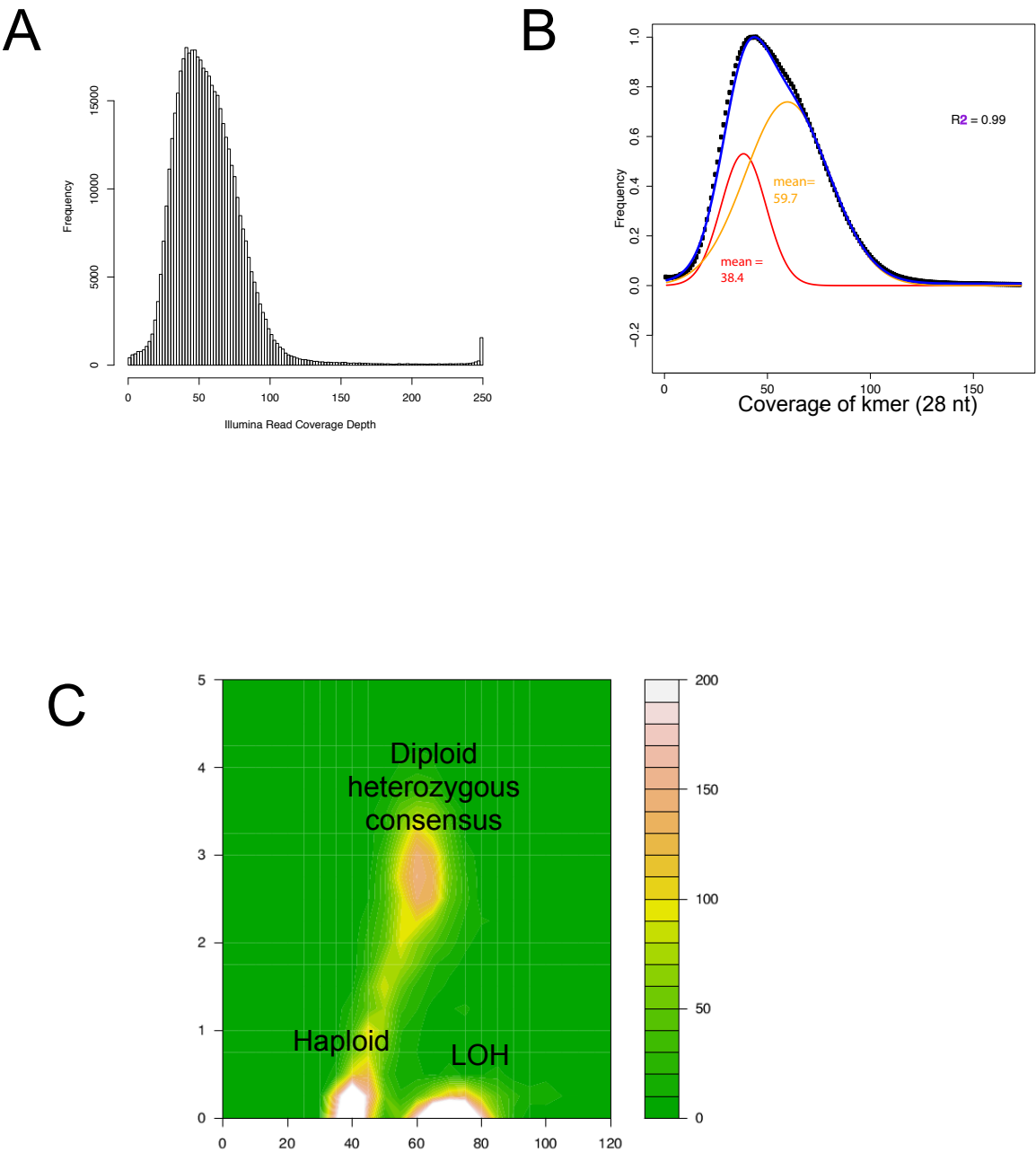

Supplement: Figure S1 — (A) Illumina read coverage of the assembly. The coverage was examined at 100 base intervals, yielding peak coverage of approximately 50× (B) Frequency of nucleotide positions with given k-mer coverage. The mean k-mer coverage (38.4) of haplotype alleles was calculated from the first Gaussian curve (colored red). The mean k-mer coverage (59.7) of the single copy sequences was calculated from the second Gaussian curve (colored orange) fitted to the main peak. The single copy sequences' coverage was used to calculate the total genome size. Relative abundance from 0 to 1 of nucleotides was plotted. The fitted Gaussian curves have R2 >0.999 (P<1e-10). (C) Illumina read coverage and polymorphism rates averaged across non-overlapping 5 kb genomic regions. Color bar indicates numbers of 5 kb regions. The plot shows partitioning of the genome segments into three groups: separated haplotypes, diploid homozygous involving LOH, and diploid heterozygous (as defined in Text S1). (PDF) [file pgen.1003272.s001.pdf]

# Supplementary Figure S2

A

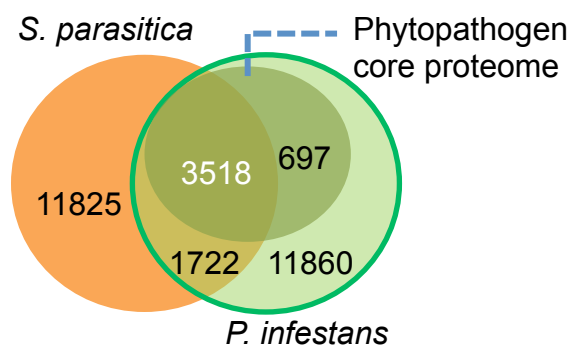

B

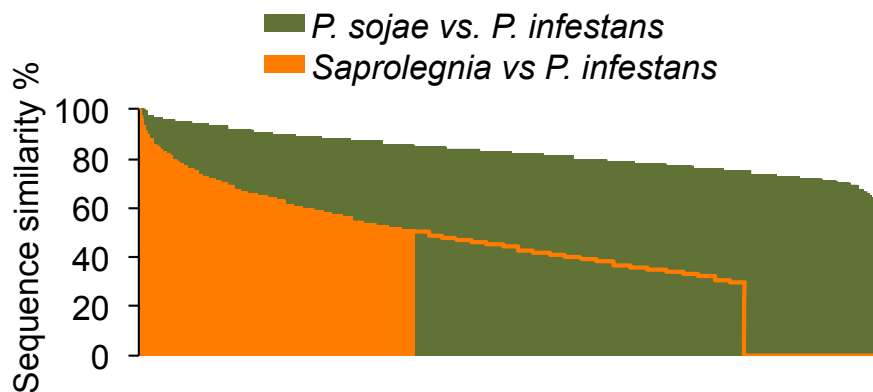

Supplement: Figure S2 — Gene content differences between S. parasitica and Phytophthora. (A) Number of genes orthologous between S. parasitica and P. infestans. The core proteome that is conserved among multiple Phytophthora species is indicated with a dark green circle. The phytopathogen core proteome derived from P. infestans, P. ramorum, P. sojae, Pythium ultimum, and Hyaloperonospora arabidopsidis. (B) Core proteome differences between S. parasitica and Phytophthora. Core protein sequences from P. sojae (green) and S. parasitica (orange) are ordered by their amino acid similarity to orthologous P. infestans proteins. Sequences with high similarity (>50%) are shown in solid, while those with less similarity (between 50% and 30%) are shown with a line. Sequences with less than 30% are not shown. (PDF) [file pgen.1003272.s002.pdf]

# Supplementary Figure S3

A

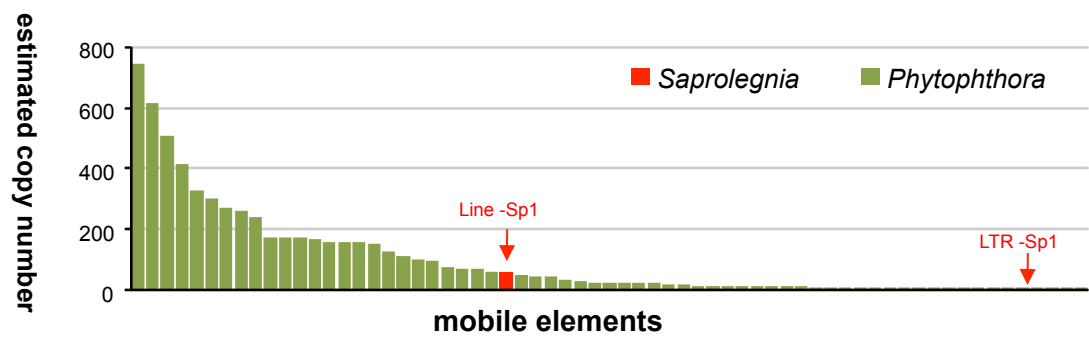

B

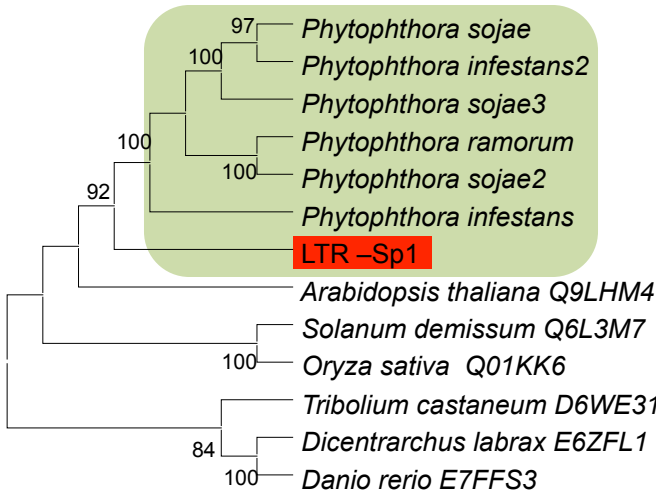

C

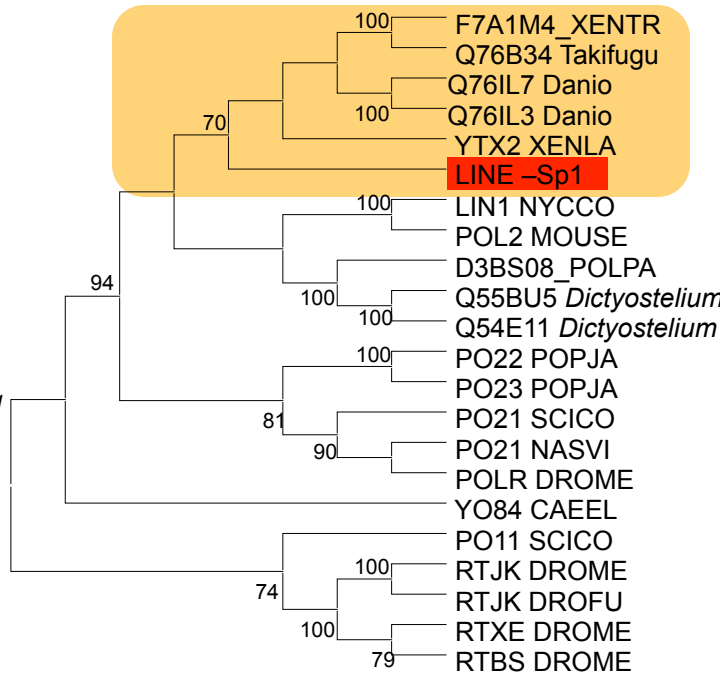

Supplement: Figure S3 — Mobile element comparison between S. parasitica and Phytophthora. (A) Mobile elements in S. parasitica and Phytophthora. The average copy number in P. infestans, P. sojae and P. ramorum is used as the copy number for Phytophthora. The elements are sorted based on the estimated copy number. (B) The S. parasitica element LTR-Sp1 is similar to the Copia-like family (Q572G9_PHYIN) in Phytophthora species. (C) The S. parasitica line element Line-Sp1 shows most homology with LINE elements found in fish and amphibian species (no other similar elements were found in other animal species). SwissProt protein species codes were used to name the sequences. The phylogenetic tree was constructed by using the neighbor joining method with 5000 replicates for bootstrap analysis. (PDF) [file pgen.1003272.s003.pdf]

# Supplementary Figure S4

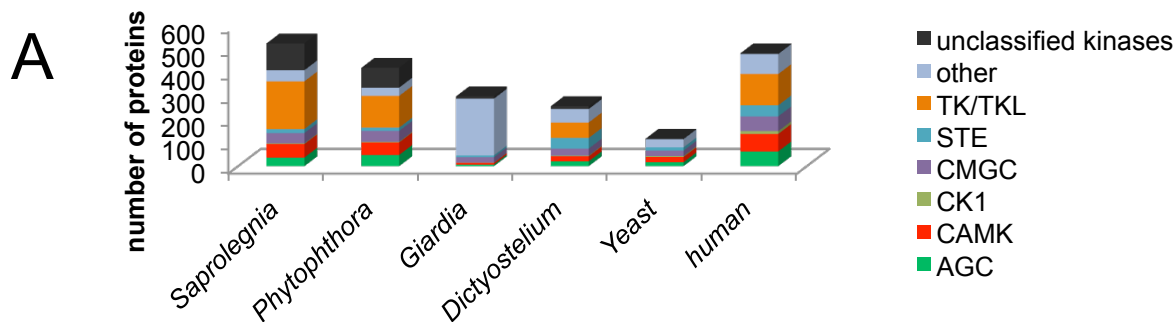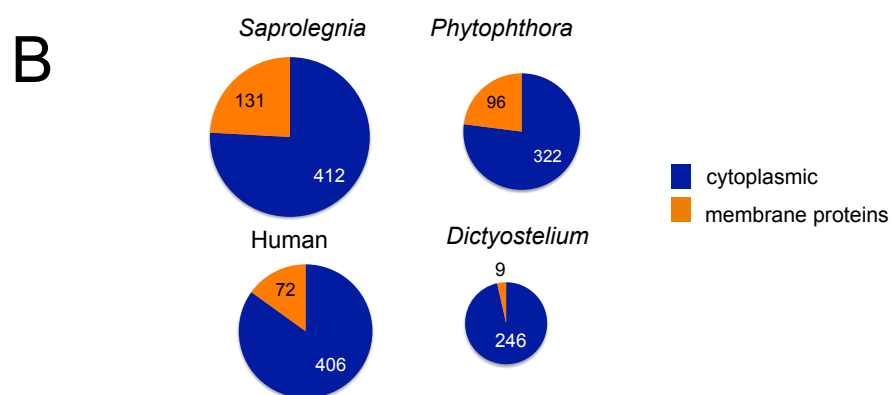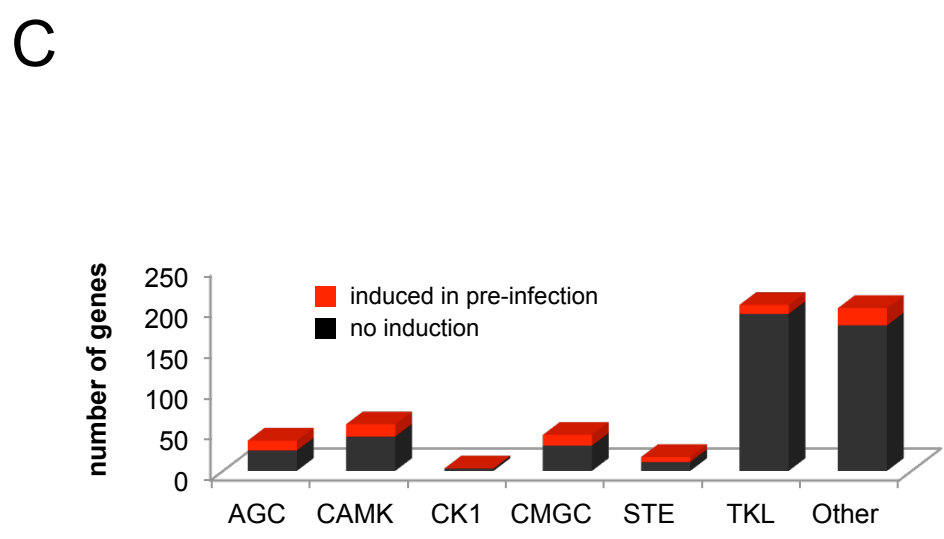

Supplement: Figure S4 — The expanded kinome of S. parasitica. (A) The distribution of S. parasitica kinases compared to other organisms. The kinases are named after the Standard Kinase Classification Scheme at kinase.com. TK = tyrosine kinase; TLK = TK-like; STE = STE7,11,20 family of MAP kinases; CMGC = (CDK, MAPK, GSK3 and CLK) family; CK1 = cell (casein) kinase 1 family; CAMK = Calmodulin/Calcium modulated kinase family; AGC = Protein Kinase A, G, and C families. The unclassified kinases are indicated in black. (B) S. parasitica contains a large number of protein kinases that contain trans-membrane helices. Pies are scaled to the total number of kinases in each species. (C) Kinase genes that are induced in the germinating cyst stage in S. parasitica compared to mycelia. Transcripts elevated more than four-fold relative to vegetative stages are considered to be induced. (PDF) [file pgen.1003272.s004.pdf]

# Supplementary Figure S5

A

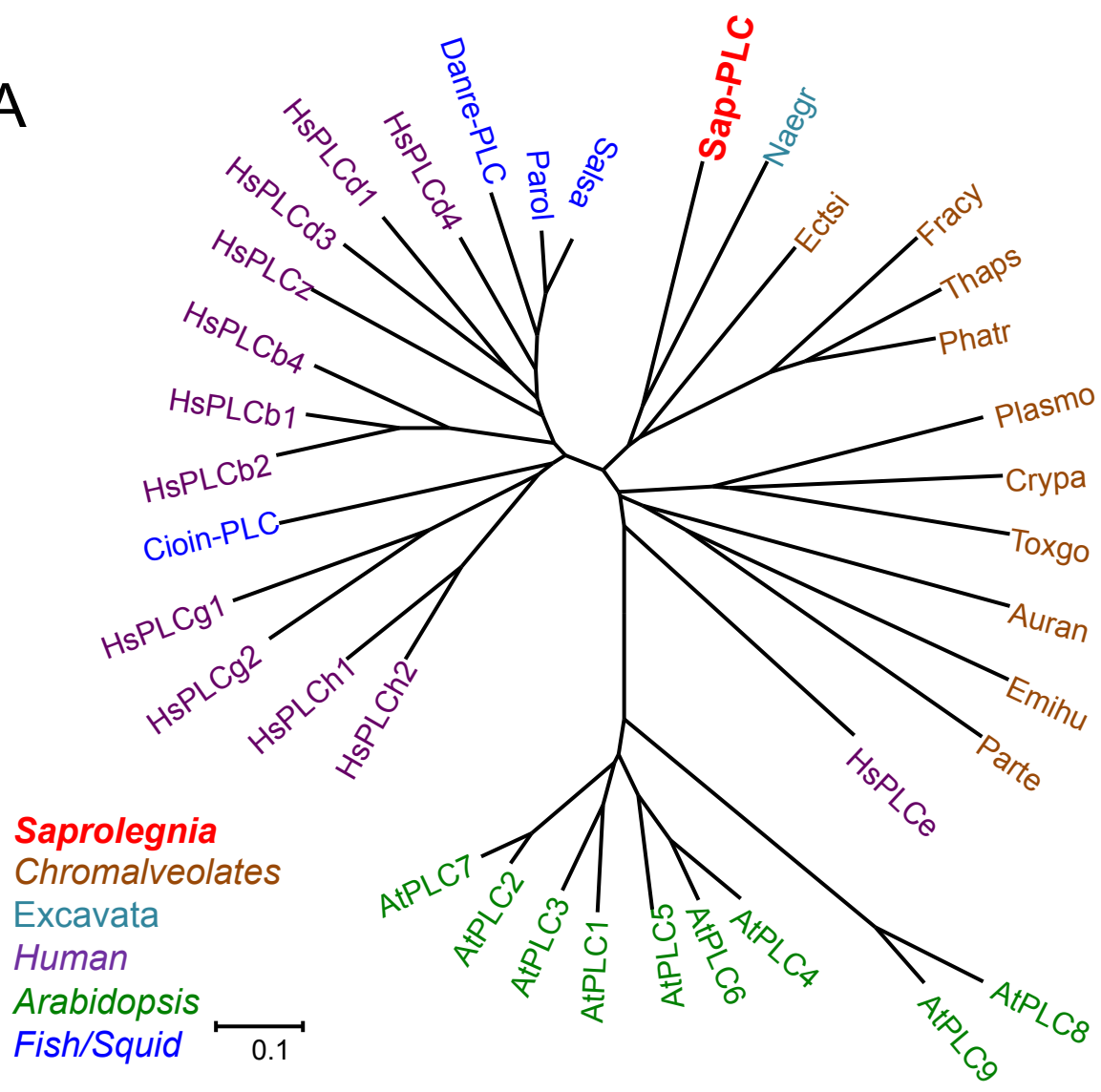

B

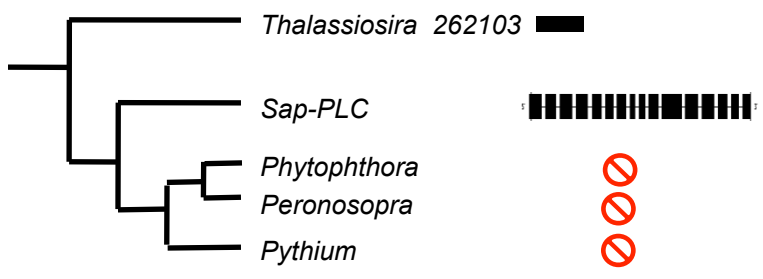

Supplement: Figure S5 — (A) Phylogram of PLCYc domains of S. parasitica PLC1 and PLCs from various organisms. For phylogenetic analysis, the PLCYc domains were determined by Smart (http://smart.embl-heidelberg.de), alignments were made and regions containing gaps were eliminated resulting in a total of 88 positions in the final dataset. The optimal tree was inferred using the Neighbor-Joining method with 5000 replicates and constructed using MEGA version 4. PLC sequences were derived from NCBI (*), JGI databases (http://genome.jgi-psf.org/,#), the Sanger Institute (http://www.genedb.org,), http://bioinformatics.psb.ugent.be,). Arabidopsis thaliana AtPLC1 (Q39032*), AtPLC2 (Q39033*), AtPLC3 (Q56W08*), AtPLC4 (Q944C1*), AtPLC5 (Q944C2*), AtPLC6 (UPI000034EE4D*), AtPLC7 (Q9LY51*), AtPLC8 (Q9STZ3*), AtPLC9 (Q6NMA7*); Aureococcus anophagefferens (Auran; 18506#); Ciona intestinalis (Cioin; XP_002129990*); Cryptosporidium parvum (Crypa; Q5CR08*); Danio rerio (Danre; XP_689964*); Ectocarpus siliculosus (Ectsi; Esi0000_0131$); Emiliania huxleyi (209393#); Fragilariopsis cylindrus (Fracy;186252#); Homo sapiens (as described by [9]*); Naegleria gruberi (Naegr; 1225#); Paralichthys olivaceus (Parol; ACA05829*); Paramecium tetraurelia, (PLC1, see [10]*); Phaeodactylum tricornutum (Phatr; 42683#), Plasmodium falciparum (Plasmo; PF10_0132@); Salmo salar (Salsa; NP_001167177*); S. parasitica: Sap-PLC (SPRG_04373#), Thalassiosira pseudonana (Thaps; 263246#), Toxoplasma gondii (Toxgo; XP_002367229*). (B) Gene structure of PLC genes. PLC is missing from other sequenced oomycete genomes, but present in S. parasitica. Multiple introns have been identified in the S. parasitica PLC gene. (PDF) [file pgen.1003272.s005.pdf]

# Supplementary Figure S6

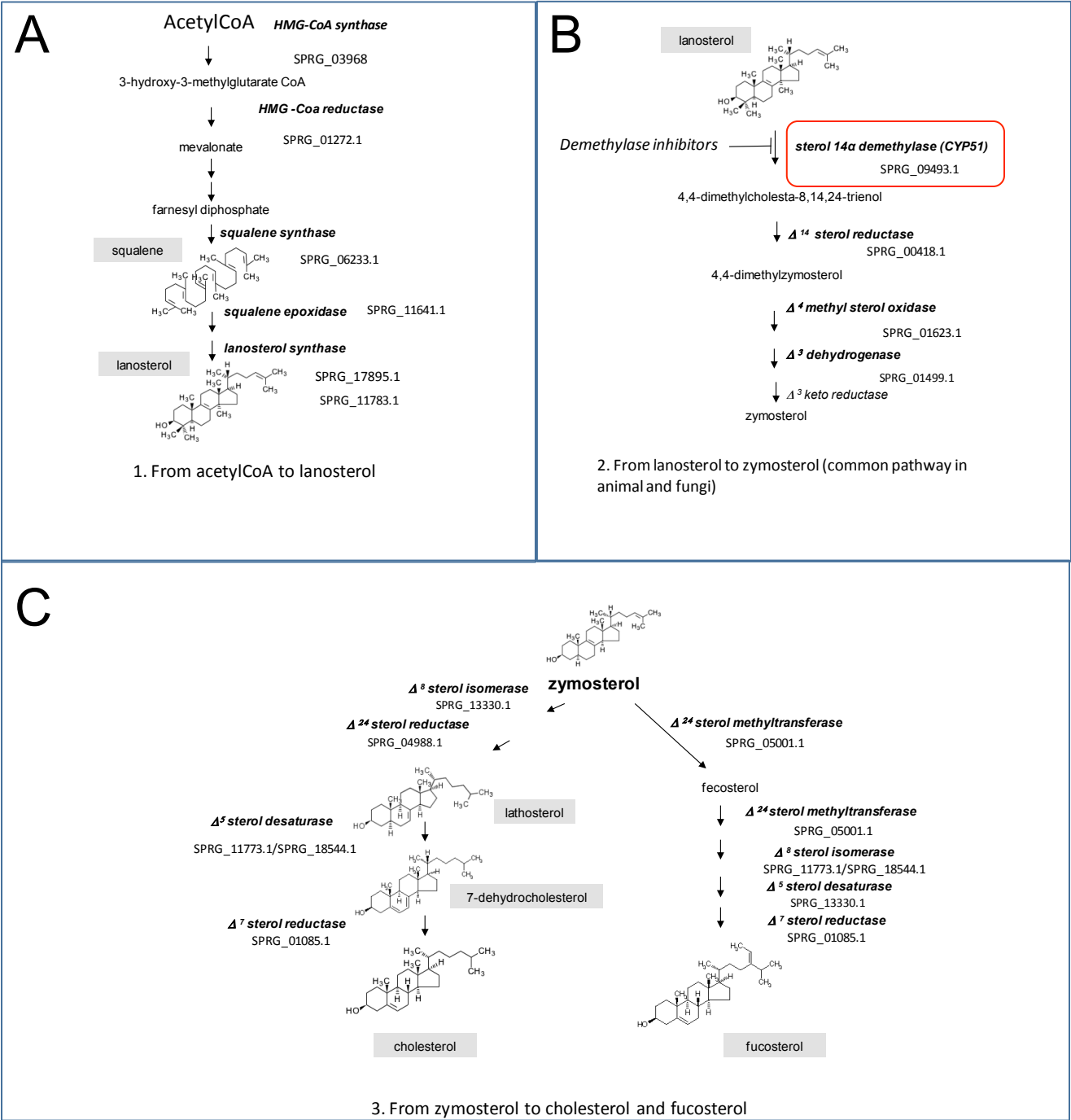

Supplement: Figure S6 — Sterol biosynthetic pathway inferred in S. parasitica. (A) The pathway from acetyl-CoA to lanosterol. (B) The pathway from lanosterol to zymosterol. The red box shows CYP51 sterol demethylase, a target of azole anti-fungal chemicals. (C) Pathways from zymosterol to cholesterol and fucosterol. (PDF) [file pgen.1003272.s006.pdf]

# Supplementary Figure S7

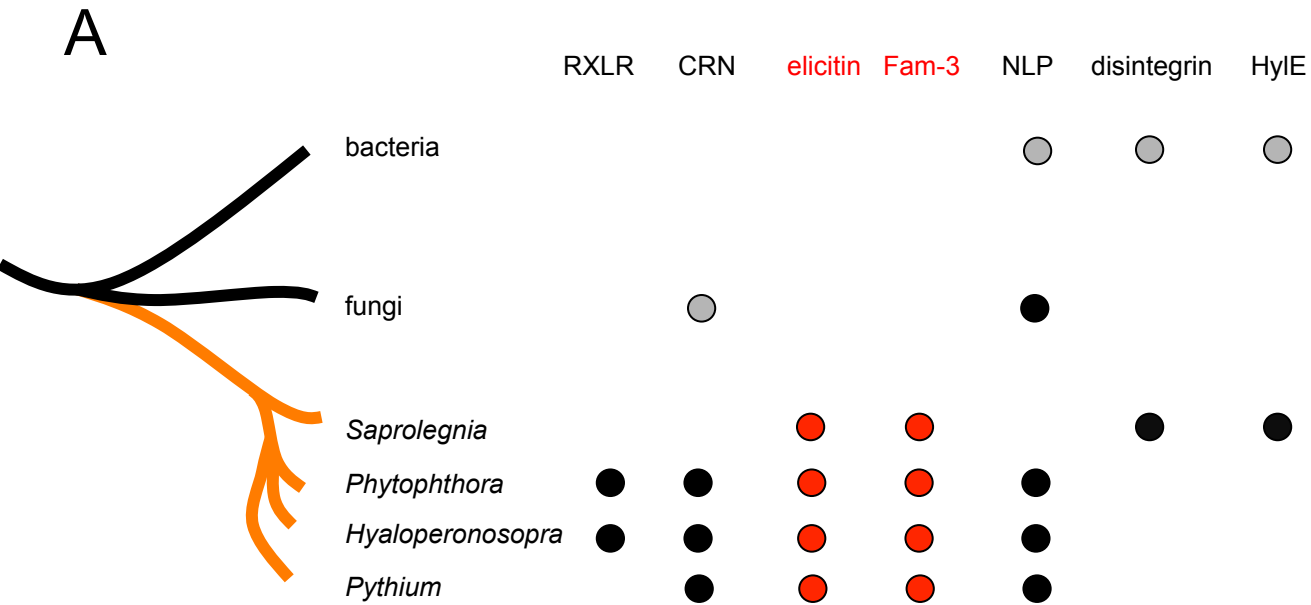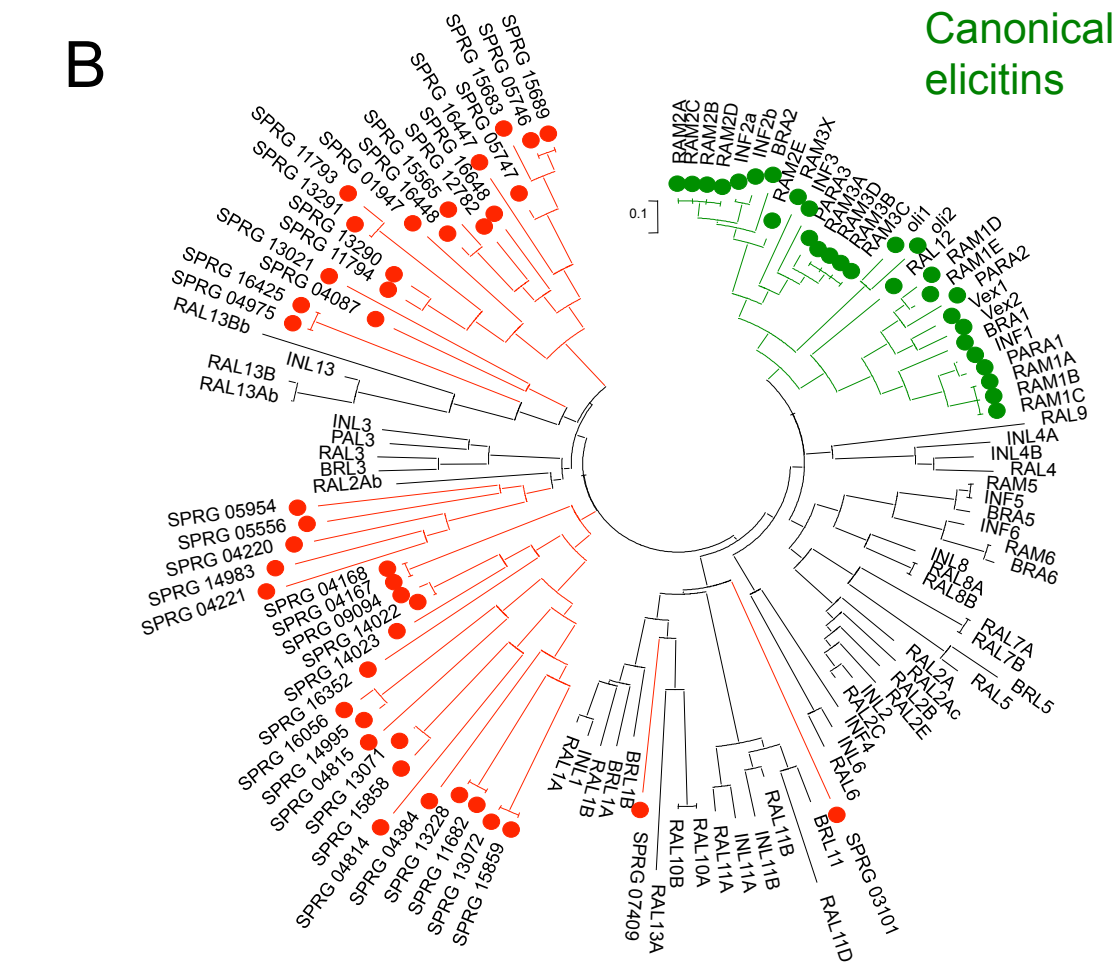

Supplement: Figure S7 — Phylogenetic distributions of infection-related molecules. (A) Classes of infection-related molecules. Two groups of PAMPs, elicitin-like and cys-rich-family-3 proteins are present in both animal- and plant-pathogenic oomycetes (colored red). The gray dots indicate infrequent occurrences. (B) Elicitin-like proteins in S. parasitica and Phytophthora. The canonical Phytophthora and Pythium elicitins are colored green. S. parasitica elicitin-like proteins are divergent and form species-specific clades. (PDF) [file pgen.1003272.s007.pdf]

# Supplementary Figure S8

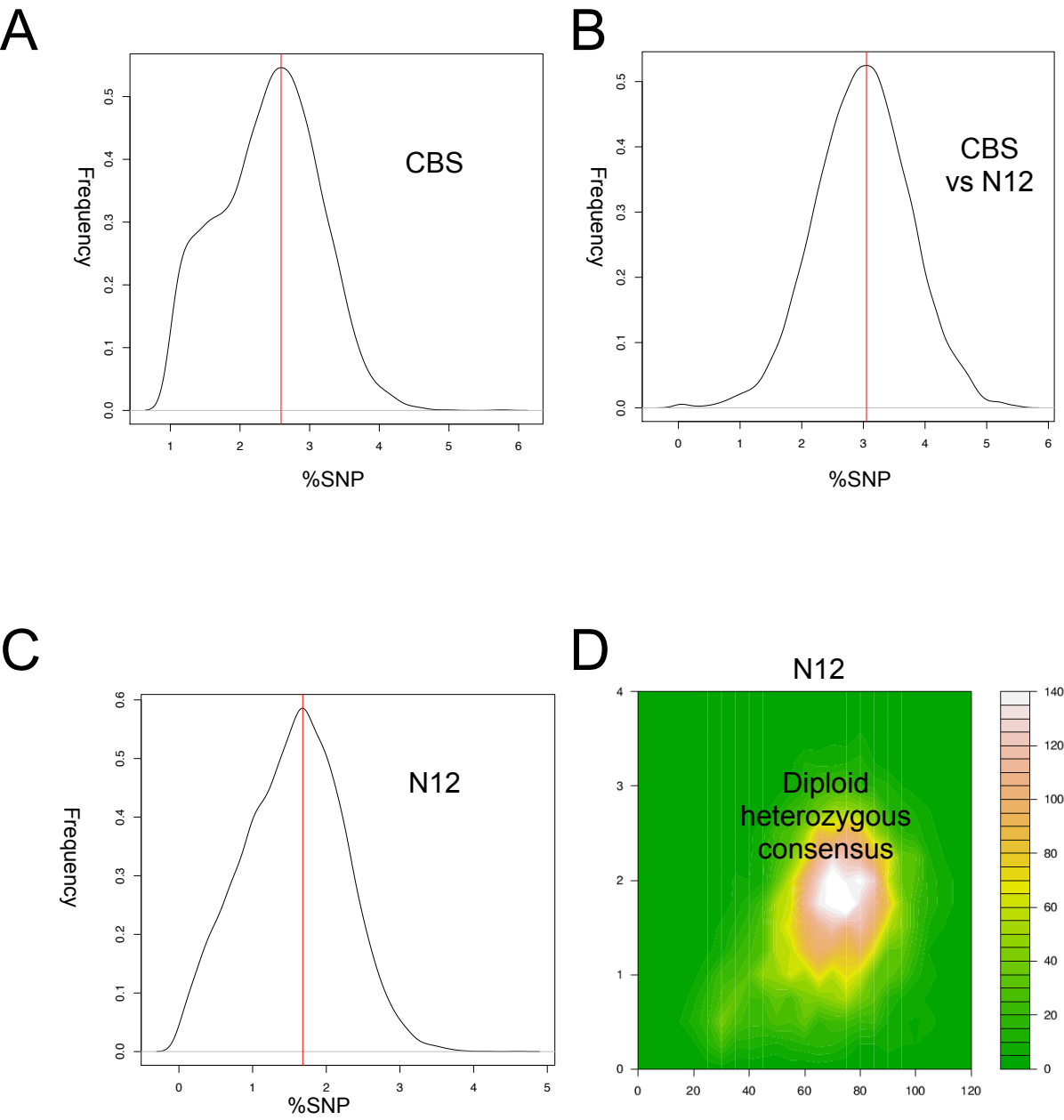

Supplement: Figure S8 — Distribution of rates of polymorphisms. (A) Summary of SNP content across 5 kb regions for Saprolegnia CBS and N12 strains. (B) Density of SNPs according to 5 kb regions of the CBS genome. The mode for the SNP rate is 2.6%. The bulge on the left side of the distribution likely corresponds to 5 kb regions of the assembly that are mosaic between haplotype and consensus diploid, as can be seen having overlap in the distribution shown in the contour plot (Figure S1C). (C) Distribution of rates of polymorphisms between strains CBS and N12. Both heterozygous and homozogous polymorphic sites were considered across 5 kb regions of the CBS genome with Illumina reads aligned from strain N12. The mode for the %SNP was computed as 3.1%. (D) Distribution of rates of polymorphisms within strain N12. Only heterozygous sites were examined in the alignments of Illumina N12 reads to the CBS strain's genome. The mode for the %SNP was computed to be 1.7%. (PDF) [file pgen.1003272.s008.pdf]
