# Supplementary material for: Distinctive Expansion of Potential Virulence Genes in the Genome of the Oomycete Fish Pathogen Saprolegnia parasitica
Source: PLoS Genet. 2013 Jun 13;9(6):e1003272. doi: 10.1371/journal.pgen.1003272 (PMC3681718; doi:10.1371/journal.pgen.1003272)

Supplementary Figure S9

A

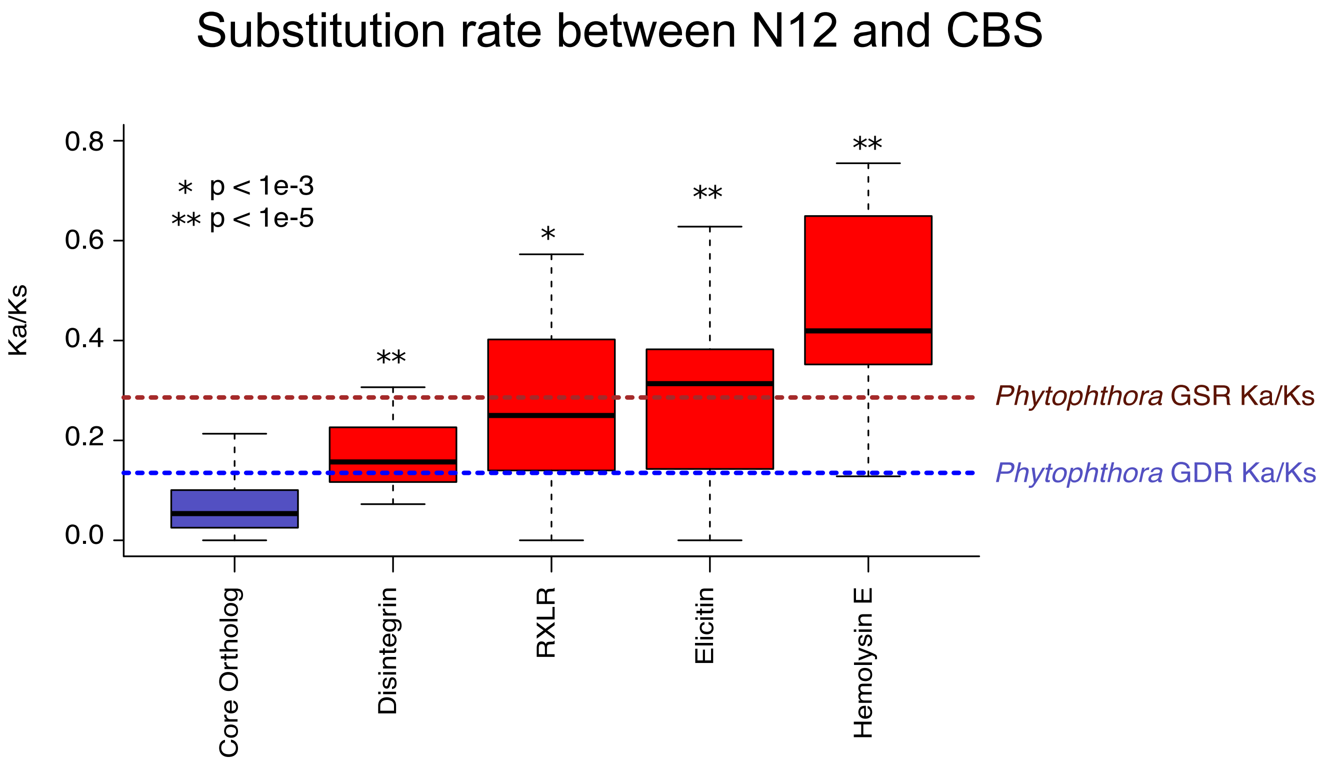

B

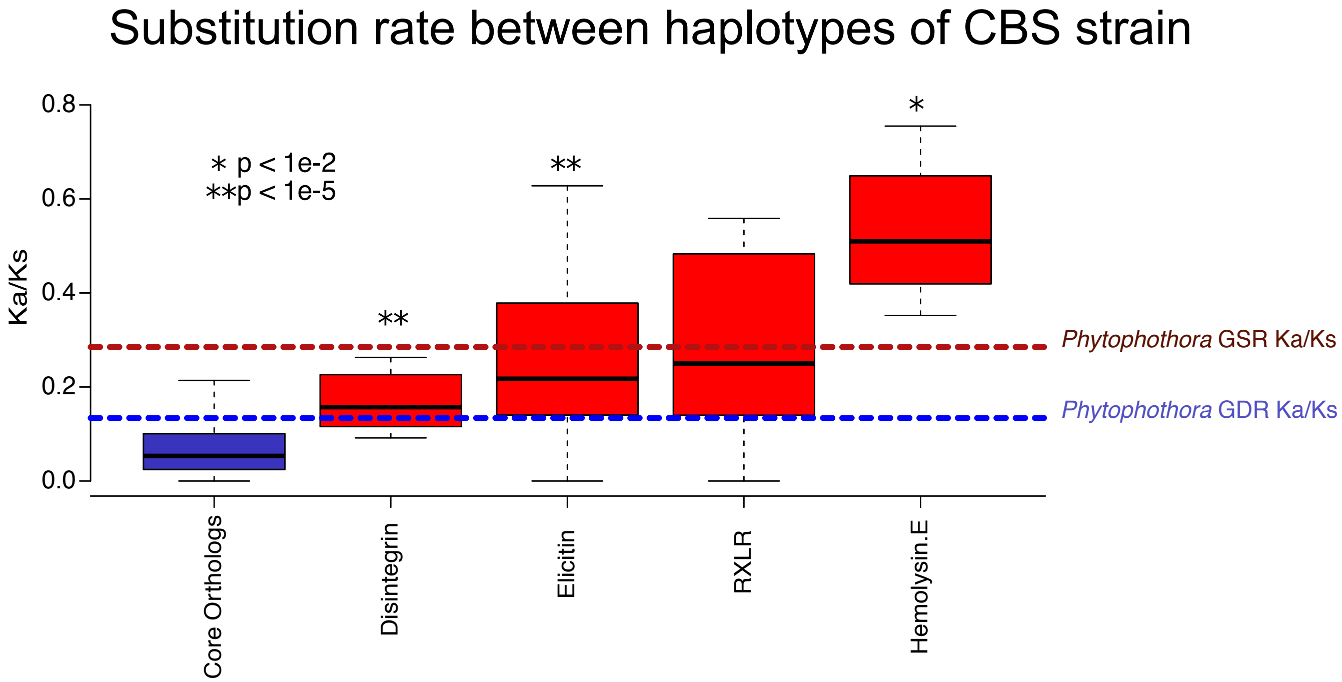

Supplement: Figure S9 — (A) Nucleotide substitution rate between S. parasitica strain CBS223.65 and N12. Asterisks indicate significant differences between the gene family and the core orthologs (* p<0.001; ** p<10−5) based on a non-parametric Z-test. Phytophthora data is based on the published results of Raffaele et al. (2010). GSR (Gene Sparse Region), GDR (Gene Dense Region). (B) Nucleotide substitution rate between the separated haplotypes of the strain of S. parasitica strain CBS223.65. (PDF) [file pgen.1003272.s009.pdf]
