# Supplementary material for: Distinctive Expansion of Potential Virulence Genes in the Genome of the Oomycete Fish Pathogen Saprolegnia parasitica
Source: PLoS Genet. 2013 Jun 13;9(6):e1003272. doi: 10.1371/journal.pgen.1003272 (PMC3681718; doi:10.1371/journal.pgen.1003272)

Supplementary Figure S10

A

MDYVIDRMPVCVTIAHCPILPCHDLVACSTTGHCQYTKHTKGTLCPSQGCSNGGFCDDDANDACDADGECVDGFFPSTYV**CRWPLGDCDVPELC**T
GYSGTCPDDALAAFGAPCTGIANGAPCDGQDICDGNGACIDQLEPAGAMCNGDVEANPCIIPGTCCGTTRTCSAPRKARVGTPCTGESQGQVCDA
PDTCDGDGRCVDRFEHGTICKIALDYSRAVFCNGRTGACPVSSFMEASDVETTGNEVAKASAETSSNVLVAHSSPSLLVVGVIGVVVGGIAVAAY
MRQSNVTPGDYVALSLMNSSSVD**MASSEDVIKEFMRFKVRMEGSVNGHEFEIEGEGEGRPYEGTQTAKLKVTKGGPLPFAWDILSPQFQYGSKAY**
VKHPADIPDYLKLSFPEGFKWERVMNFEDGGVVTVTQDSSLQDGEFIYKVKLRGTNFPSDGPVMQKKTMGWEASTERMYPEDGALKGEIKMRLKL
KDGGHYDAEVKTTYMAKKPVQLPGAYKTDIKLDITSHNEDYTIVEQYERAEGRHSTGAKAAALEHHHHHH

B

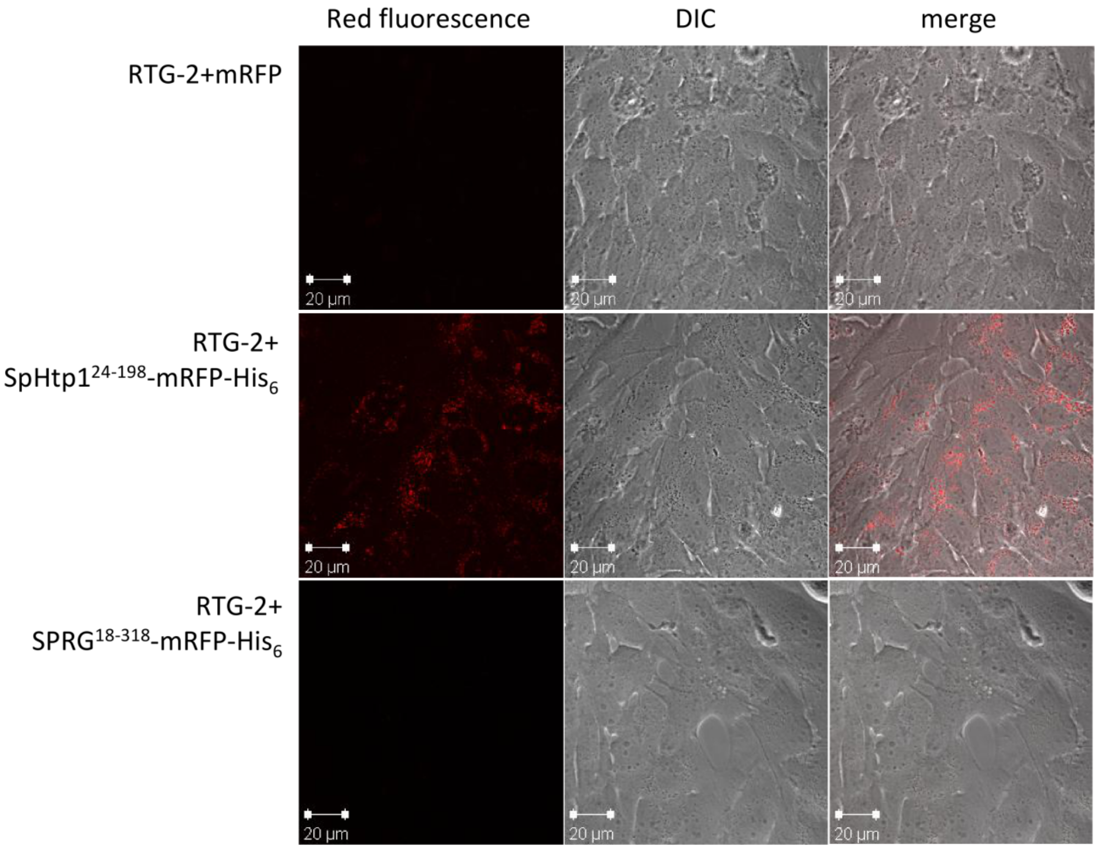

Supplement: Figure S10 — Predicted disintegrin SPRG_14052 does not enter fish cells in vitro. (A) Amino acid sequence of fusion protein SPRG_14052_mRFP-His6. The CRxxxxxCDxxExC disintegrin motif is shaded in red. The mRFP sequence is indicated in blue, the His-tag is in green. (B) RTG-2 cells were exposed to 3 µM of mRFP, SpHtp1 or SPRG_14052_mRFP-His6 and incubated for 30 min, before photography. (PDF) [file pgen.1003272.s010.pdf]
