# Supplementary material for: Distinctive Expansion of Potential Virulence Genes in the Genome of the Oomycete Fish Pathogen Saprolegnia parasitica
Source: PLoS Genet. 2013 Jun 13;9(6):e1003272. doi: 10.1371/journal.pgen.1003272 (PMC3681718; doi:10.1371/journal.pgen.1003272)

Supplementary Figure S11

A

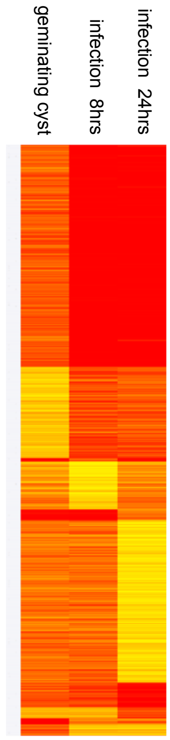

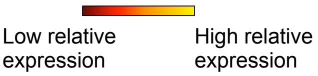

B

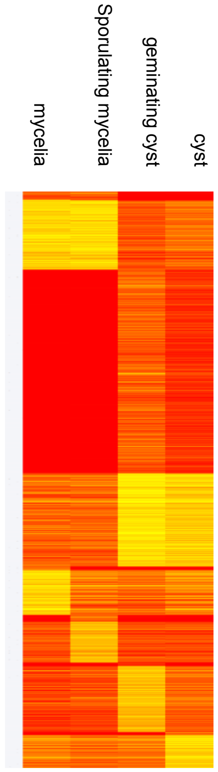

Differentially expressed genes during the infection time course

Differentially expressed genes in different life stages

C

	my	s_my	cy	g_cy	8hrs	24hrs
my	1					
s_my	0.88	1				
cy	0.77	0.74	1			
g_cy	0.84	0.79	0.93	1		
8hrs	0.85	0.79	0.87	0.88	1	
24hrs	0.43	0.36	0.34	0.39	0.44	1

D

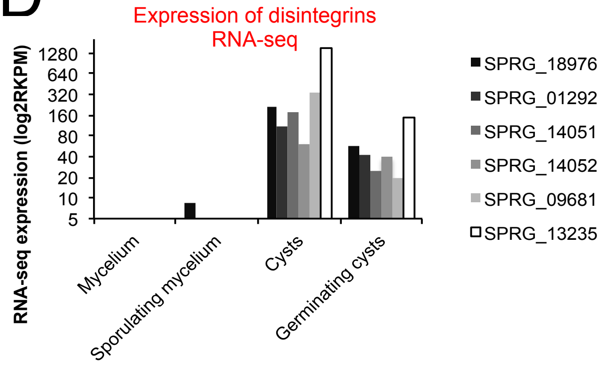

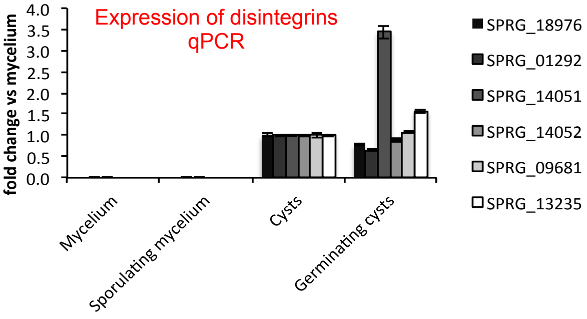

Supplement: Figure S11 — Stage specific gene expression detected by RNA-Seq in the total gene set. (A) Genes differentially expressed during fish cell interaction. (B) Differentially expressed genes in different life stages. (C) The correlation coefficients of pairwise comparisons between RNA-Seq data sets (p<0.001). (D) Transcript levels of a subset of disintegrin-encoding genes in various life stages of S. parasitica determined by RNAseq and qPCR. For RNAseq, the log2 value of RKPM of a gene is plotted. For qPCR, transcript levels are relative to the transcript levels of SpHtp1 in cysts and normalized against the reference gene SpTub-b encoding for tubulin. Error bars correspond to four biological replicaties. (PDF) [file pgen.1003272.s011.pdf]
