# Supplementary material for: Distinctive Expansion of Potential Virulence Genes in the Genome of the Oomycete Fish Pathogen Saprolegnia parasitica
Source: PLoS Genet. 2013 Jun 13;9(6):e1003272. doi: 10.1371/journal.pgen.1003272 (PMC3681718; doi:10.1371/journal.pgen.1003272)
Supplement: Text S1 — Supplementary information on: phospholipid modifying enzymes and signaling enzymes, sterol metabolism, disintegrin-like proteins and supplementary methods. (DOCX) [file pgen.1003272.s012.docx]

**Text S1 for:**

**Distinctive expansion of potential virulence genes in the genome of the oomycete fish pathogen *Saprolegnia parasitica***

**Phospholipid modifying and signalling enzymes**

Oomycetes have a core set of phospholipid modifying and signalling enzymes (PMSE) which in many aspects differs from the set found in other eukaryotes (Meijer and Govers, 2006; Meijer *et al.*, 2011). The core set of PMSE is conserved between animal and plant pathogenic oomycetes. The *S. parasitica* genome has a total of 55 genes that encode PMSE, including phosphatidylinositol synthase (PIS), phosphatidylinositol kinase (PIK), phosphatidylinositolphosphate kinase (PIPK), diacylglycerol kinase (DGK) and phospholipase D (PLD) (**Supplementary TableS6**). The most striking difference between *S. parasitica* and other known oomycetes is the presence of a phospholipase C (PLC) gene. *S. parasitica* contains a gene encoding phospholipase C (PLC), which is absent for all other oomycetes investigated to date. The PLC gene has 14 introns, which suggests it has been retained in *S. parasitica* from the stramenopile ancestor rather than horizontally acquired from other species. Phylogenetic analysis groups the *S. parasitica* PLC gene with that of the heterokont species (**Supplementary Fig. S5B**), therefore we conclude the *S. parasitica* PLC is mostly likely to be ancient and the absence of PLC in other oomycetes is due to gene loss in these lineages.

**Sterol metabolism**

Sterols are isoprenoid compounds playing an essential role in eukaryotic cells, controlling membrane fluidity and permeability and being precursors of steroid hormones in mammals, plants and oomycetes. However, oomycete species belonging to the peronosporomycetidae are sterol auxotrophs and genomic studies have revealed that most genes encoding enzymes involved in sterol biosynthesis are missing in these organisms (Gaulin *et al*., 2010). In contrast, sequencing of an EST collection and biochemical analyses revealed that *Aphanomyces euteiches*, in the saprolegniomycetidae has a new sterol biosynthetic pathway (Madoui *et al*, 2007). A complete set of orthologous sterol biosynthesis genes was found in the *S. parasitica* genome by searching with the *Aphanomyces* genes (**Supplementary Fig. S6**). It is mostly likely that fucosterol and cholesterol are major end-sterols in *S. parasitica*. Importantly, a gene coding the CYP51 sterol-demethylase has been identified (**Supplementary Fig. S6**). This protein is a major target of antifungal chemicals, which could perhaps also be used against pathogens in the saprolegniomycetidae.

**Biochemical analysis of predicted disintegrin family proteins**

The *S. parasitica* disintegrin family consists of 16 putative proteins, of which 4 are predicted to be soluble while the others contain transmembrane domains. One disintegrin protein, SPRG_14052 (**Supplementary Fig. S10A**), was selected to determine if it had the ability to affect the physiology of fish cells or the ability to enter them. The protein was expressed in *E. coli* as a fusion to mRFP with a C-terminal his tag. When this protein was purified and exposed either to RTG-2 cells, to *O. mykiss* RTL-W1 cells (Lee *et al.*, 1993) or to *O. mykiss* red blood cells or platelets, no observable changes were observed in the physiology of the cells. Furthermore, no entry by the proteins into any of the cells was detected (**Supplementary Fig. S10B**). In contrast, SpHtp1-mRFP could be readily observed inside RTG-2 cells (**Supplementary Fig. S10B**). SPRG_14052^19-318^-mRFP-His_6_ was also tested on *O. mykiss* blood cells, but no effect on blood cell number or morphology was observed (data not shown). Also, no binding to or translocation of SPRG_14052^19-318^-mRFP-His_6_ into red blood cells or platelets was observed (data not shown).

**Supplementary Methods**

**Protein expression**

For expression of SPRG_14052, a synthetic construct for over-expression was ordered from Genscript Inc (Boston), excluding the signal peptide and including NdeI and EcoRI restriction sites. Each construct was subcloned from the provided vector pUC57 into pET21b by restriction with NdeI and EcoRI, producing a construct (SPRG_14052^19-318^-mRFP-His_6_) encoding a fusion with mRFP and His tag under the control of the T7 promoter (Wawra *et al.,* 2012). For protein expression, *E. coli* Origami B(DE3)pLysS cells (Novagen) carrying the construct were grown in LB-media to an OD_600_ between 0.6 and 0.8, then induced with 1mM IPTG for 6 hr at 37°C. After centrifugation, the cell pellet was re-suspended in 40 ml 25 mM sodium-phosphate (NaP_i_) pH 7.5 and incubated for 20 min with 250 U of Benzonase (Sigma), two tablets of protease inhibitor (Roche, #11873580001) and 0.1 g lysozyme (Fluka, #62971). After the incubation, the suspension was disrupted in a French press, diluted 1:5 in the respective buffer and the soluble fraction was recovered by centrifugation at 50,000xg for 1 hr. The French press supernatant was passed onto a sulfate column (40 ml Fractogel-EMD-SO3^-^ (M) (Merck, #1.16882.0100), adjusted with 25 mM NaP_i_ pH 7.5 and was washed with 5 volumes of the same buffer. The flow through was passed onto a NTA column (15 ml NTA Agarose; Invitrogen, #60-0441), and washed with 25 mM NaP_i_ containing 30 mM imidazole (pH 7.5). The protein was eluted from the column with 25 mM NaP_i_ containing 300 mM imidazole (pH 7.5) and fractions were analyzed with SDS-PAGE (4-12% Bis-Tris, MOPS running buffer; Invitrogen). Protein samples were dissolved in Laemmli buffer containing 8M urea and 2% β-mercaptoethanol. The expected protein size was 58.4 kDa. All fractions of pure protein were dialyzed once for 3 hr and once overnight against 25 mM NaP_i_ PH 7.5.

**Protein Cell entry assays.**

RTG-2 cells were maintained at 24 °C in 25 cm^2^ or 75 cm^2^ tissue culture flasks (Nunc) in supplemented L15 medium. Fibroblast epithelial cell line RTL-W1, derived from *O. mykiss* liver cells (Lee *et al.*, 1993), were maintained as for the RTG-2 cells. The fish cell lines were exposed to 3 and 10 µM SPRG_14052^19-318^-mRFP-His_6_ for 30 min, 2.5 hr or 24 hr in similar way as described before for SpHtp1 (Wawra *et al.,* 2012). Images were recorded using a Zeiss LSM510 confocal microscope equipped with a water dipping lens. All images were obtained using the same microscope settings for all treatments. The microscope settings were: optical slice = 2 μm, Red channel: Excitation: 543 nm; Detector gain: 750; Filter setting: LP 560 nm. None of the concentrations showed binding or translocation of µM SPRG_14052^19-318^-mRFP-His_6_ for all incubation lengths tested (**Supplementary Fig. S10**).

**Putative Ricin B-like lectins from *S. parasitica***

We wanted to investigate whether the ricin_B_like lectin domain, found in several putatively secreted proteases, can facilitate the uptake of the protease domain into host cells. Therefore protein over-expression constructs were made for one candidate gene (SPRG_21856.2) to generate recombinant ricin_B proteins to be tested in fish cell uptake experiments as previously described (Wawra *et al*., 2012). Gene expression data showed that the SPRG_21856.2 gene was expressed throughout the life stages of *S. parasitica* with a higher expression level in cysts and germinating cysts compared to the other life stages. For studying protein translocation into fish cells three constructs of SPRG_21856.2 were generated: 1) full-length (protease + ricin-B-like domain); 2) full-length with a mutation in the putative protease domain; and 3) truncated with only the putative ricin-B-like domain. All constructs were C-terminally fused to the red fluorescence protein mRFP and over-expressed in Origami 2 *E. coli* cells. For both full-length constructs no over-expression was observed in *E. coli*. For the ricin B-like domain over-expression was possible, however the protein was always degraded upon purification. Also a non mRFP-fused variant of the ricin B-like domain could not successfully be purified. Changing the expression system to *Pichia pastoris*.did not help, unfortunately none of the recombinant proteins could successfully be purified due to degradation. Furthermore a construct with the mRFP fused N-terminally to the Ricin B-like domain did also result in degradation of the recombinant protein. We were therefore unable to demonstrate a possible role for the ricin_B like lectin domain found in several putative *S. parasitica* proteins.

**Protein kinases identification**

Protein kinases were identified by searching their proteins against a protein kinase HMM derived from an alignment of *Dictyostelium* protein kinases (Goldberg et al., 2006) using a cutoff score of -66. Low-scoring sequences were additionally screened for conservation of known protein kinase sequence motifs. The kinases thus identified were classified using the system of Hanks and Hunter (1995) and Manning and coworkers (2002).

**Estimating collapsed repeat occurrence and genome size from the read coverage**

The Illumina read coverage of the assembly was examined at 100 base intervals (coverage distribution shown in **Supplementary Fig. S1A**), yielding peak coverage of approximately 50X. Local copy number was estimated for each interval based on the floor (observed_coverage/50), and the number of bases collapsed in the assembly was computed by summing up the product of the number of bases in intervals and the estimated copy numbers exceeding 1. The total number of bases estimated to be collapsed in the assembly is 20,439,888.

We have used the total amount of sequencing reads divided by the single copy sequences’ coverage to calculate the total genome size (**Supplementary Fig. S1B**). The mean *k*-mer coverage (59.7) of the single copy sequences was calculated from a Gaussian curve that was fitted to the main peak of the coverage distribution (R^2^ > 0.999, p<1e-10). The estimated genome size of *S. parasitica* CBS strain based on repeat analysis is ~62 Mb.

**Estimating the effective sequenced genome size considering separately assembled haplotypes and collapsed repeats.**

The assembled *Saprolegnia* genome of cumulative contig length of 48,138,513 represents a single dimensional view of the genome. By considering additional dimensions including the depth of Illumina read coverage and observed polymorphisms, we identified regions of the assembly that represent separately assembled haplotypes (polymorphism-free regions at roughly half the expected depth of read coverage), homozygous consensus regions (polymorphism-free regions at the expected depth of coverage), and diploid consensus regions (polymorphism-rich regions approaching the expected depth of coverage). These regions are apparent in **Supplementary Fig. S1C**, showing the density of 5kb genomic regions according to percentage of SNPs and Illumina read coverage; note that a 5kb region of the assembly was counted only if at least 4kb was covered by Illumina reads, excluding scaffold gaps. To estimate the proportions of the assembled genome that fall into these categories, we classified each region to category as described in **Supplementary Table S2**. Since the haploid regions are being doubly counted within the assembled contig length, we must subtract half of the haploid contribution (equal to 5,776,622 bases) to arrive at a more accurate estimate of the assembled diploid genome size of 42,361,891. If we add to this value the number of bases estimated to represent collapsed repeats in the assembly (see above, estimated at 20.4 Mb), our effective assembled genome size becomes 62.8 Mb, which is consistent with the genome size estimated based on k-mer abundance distribution. We estimated the copy number of the SpLINE element by matching the known SpLINE loci to the collapsed repeats regions of the genome. The collapsed repeats loci were obtained by delineating high reads coverage (ranging from 100 to 100,000) regions. Around a few hundred LINE elements were estimated to be collapsed in these regions of genome assembly.

**Assessing genome representation of expressed transcripts**

RNA-Seq data for each of the four growth stages (cysts, germinating cysts, mycelium, and sporulating mycelium) were each independently assembled in a strand-specific manner using the Trinity software (Grabherr *et al*., 2011). Assembled transcripts of at least 500 bases in length were mapped to the *S. parasitica* assembled genome sequence using GMAP (Wu and Watanebe, 2005). Those transcripts mapping to the genome by at least 50% of their length and with at least 90% identity were counted as being mapped to the genome.

**Computation of peak SNP rates for CBS and VI-02736 strains.**

The density of %SNPs was examined for 5kb regions (as defined above) using the *R* software. The peak SNP rate was identified as the mode of the %SNPs and computed using *R* (**Supplementary Table S11**). The CBS strain rate of polymorphism was computed using only the heterozygous diploid consensus regions of the genome (**Supplementary Fig. S8A**). Rates of polymorphism were computed for strain VI-02736 both in comparison to CBS (**Supplementary Fig. S8B**), considering both homozygous and heterozygous sites, and just within strain VI-02736 by examining only heterozygous sites (**Supplementary Fig. S8C and S8D**).

References

Gaulin E, Bottin A, Dumas B. (2010) Sterol biosynthesis in oomycete pathogens. Plant Signal Behav. 5: 258-260.

Goldberg JM, Manning G, Liu A, Fey P, Pilcher KE, Xu Y, Smith JL. The dictyostelium kinome--analysis of the protein kinases from a simple model organism. (2006 ) PLoS Genet. 2 (3): e38.

Grabherr MG, Haas BJ, Yassour M, Levin JZ, Thompson DA, Amit I, Adiconis X, Fan L, Raychowdhury R, Zeng Q, Chen Z, Mauceli E, Hacohen N, Gnirke A, Rhind N, di Palma F, Birren BW, Nusbaum C, Lindblad-Toh K, Friedman N, Regev A. 2011. Full-length transcriptome assembly from RNA-Seq data without a reference genome. Nat. Biotechnol. 29: 644-652.

Hanks SK, Hunter T. (1995) Protein kinases 6. The eukaryotic protein kinase superfamily: kinase (catalytic) domain structure and classification. FASEB J. 9: 576-96.

Lee, LEJ, Clemons, JH, Bechtel, DG, Caldwell, SJ, Han, KB, Pasitschniakarts, M, et al., 1993. Development and characterization of a rainbow-trout liver-cell line expressing cytochrome P450-dependent monooxygenase activity. Cell Biol.Toxicol. 9: 279–294.

Madoui MA, Gaulin E, Mathé C, San Clemente H, Couloux A, Wincker P, Dumas B. (2007) AphanoDB: a genomic resource for Aphanomyces pathogens. BMC Genomics 8: 471.

Manning G, Whyte DB, Martinez R, Hunter T, Sudarsanam S. (2002). The protein kinase complement of the human genome. Science. 298: 1912-1934.

Meijer, H. J. G., and Govers, F. (2006). Genomewide analysis of phospholipid signaling genes in Phytophthora spp.: novelties and a missing link. Mol Plant-Microbe Interact 19:1337-1347.

Meijer, H. J. G., Hassen, H. H., and Govers, F. 2011. Phytophthora infestans has a plethora of phospholipase D enzymes including a subclass that has extracellular activity. PLoS ONE 6: e17767.

Wawra S, Bain J, Durward E, de Bruijn I, Minor KL, Matena A, Löbach L, Whisson SC, Bayer P, Porter AJ et al. 2012. Host-targeting protein 1 (SpHtp1) from the oomycete Saprolegnia parasitica translocates specifically into fish cells in a tyrosine-O-sulphate–dependent manner. Proc. Natl. Acad. Sci. USA 109: 2096-2101.

Wu TD, Watanabe CK. 2005. GMAP: a genomic mapping and alignment program for mRNA and EST sequences. Bioinformatics. 21: 1859-1875.
