# Supplementary material for: Distinctive Expansion of Potential Virulence Genes in the Genome of the Oomycete Fish Pathogen Saprolegnia parasitica
Source: PLoS Genet. 2013 Jun 13;9(6):e1003272. doi: 10.1371/journal.pgen.1003272 (PMC3681718; doi:10.1371/journal.pgen.1003272)
Supplement: Table S1 — Saprolegnia parasitica genome assembly statistics. (DOCX) [file pgen.1003272.s013.docx]

| **Scaffold length** | 53.1 Mb |
| --- | --- |
| **Contig length** | 48.1 Mb |
| **Scaffold N50** | 281 kb |
| **Contig N50** | 34.5 kb |
| **Number of contigs** | 4125 |
| **Number of scaffolds** | 1442 |
| **Q40 bases** | 99.23% |

**Supplementary Table S1: *Saprolegnia parasitica* genome assembly statistics**
