# Supplementary material for: Distinctive Expansion of Potential Virulence Genes in the Genome of the Oomycete Fish Pathogen Saprolegnia parasitica
Source: PLoS Genet. 2013 Jun 13;9(6):e1003272. doi: 10.1371/journal.pgen.1003272 (PMC3681718; doi:10.1371/journal.pgen.1003272)
Supplement: Table S2 — Assembled S. parasitica genome partitioned into classes based on coverage and polymorphisms. (DOCX) [file pgen.1003272.s014.docx]

**Supplementary Table S2: Assembled *S. parasitica* genome partitioned into classes based on coverage and polymorphisms**

| **Region type** | **Definition** | **Number of 5kb regions** | **Total number of bases by region** | **Percentage of the genome assembly** |
| --- | --- | --- | --- | --- |
| Heterozygous consensus | %SNP >= 1 | 3,891 | 19,455,000 | 44% |
| Diploid consensus | 50 <= Cov <= 100 | 2771 | 13,855,000 | 31% |
| Haploid | Cov < 50 | 2084 | 10,420,000 | 24% |
| Repeat consensus | Cov > 100 | 108 | 540,000 | 1% |
