# Supplementary material for: Distinctive Expansion of Potential Virulence Genes in the Genome of the Oomycete Fish Pathogen Saprolegnia parasitica
Source: PLoS Genet. 2013 Jun 13;9(6):e1003272. doi: 10.1371/journal.pgen.1003272 (PMC3681718; doi:10.1371/journal.pgen.1003272)
Supplement: Table S4 — Chitin biosynthesis, modification and degradation in oomycetes. (DOCX) [file pgen.1003272.s016.docx]

**Supplemental Table S4 – Chitin biosynthesis, modification and degradation in oomycetes.**

| function | CAZy family | enzymatic activity | *Spa** | *Pinf* | *P soj* | *Pram* | *Pult* |
| --- | --- | --- | --- | --- | --- | --- | --- |
|  |  |  |  |  |  |  |  |
| chitin biosynthesis | |  |  |  |  |  |  |
|  | GT 2 | chitin synthase | 6 | 1 | 2 | 2 | 1 |
| chitin modification | |  |  |  |  |  |  |
|  | CE 4 | chitin deacetylase | 2 | 1 | 2 | 2 | ND |
| chitin degradation | |  |  |  |  |  |  |
|  | GH 18 | chitinase | 12 | 2 | 3 | 2 | 2 |
|  | GH 19 | chitinase | 2 | 2 | 2 | 1 | ND |
|  | GH 20 | β-hexosaminidase | 2 | 0 | 0 | 0 | 0 |
|  | GH 85 | endo-β-N-acetylglucosaminidase | 1 | 1 | 1 | 1 | ND |

*The species names are Spa- *Saprolegnia parasitica,* Pinf *– Phytophthora infestans,* Psoj *– P. sojae,* Pram *– P. ramorum,* Pult *– Pythium ultimum*.
