# Supplementary material for: Distinctive Expansion of Potential Virulence Genes in the Genome of the Oomycete Fish Pathogen Saprolegnia parasitica
Source: PLoS Genet. 2013 Jun 13;9(6):e1003272. doi: 10.1371/journal.pgen.1003272 (PMC3681718; doi:10.1371/journal.pgen.1003272)
Supplement: Table S5 — Gene encoding nitrogen and sulphur assimilation enzymes in oomycetes. (DOCX) [file pgen.1003272.s017.docx]

**Supplemental Table S5- Gene encoding nitrogen and sulphur assimilation enzymes in oomycetes**

|  | *P. sojae* | *P. ramorum* | *H. arabidopisdis* | *S. parasitica** |
| --- | --- | --- | --- | --- |
| Nitrate reductase | *Ps140563* | *Pr71442* | *none* | *none* |
| Nitrite reductase | Ps140562 | Pr76696 | none | none |
| Nitrate transporter3 | Ps140564 | Pr76698 | none | none |
| Glutamine synthetase | Ps109139 | Pr72153 | Ha802420 | SPRG_06768  SPRG_02883 |
| Glutamate synthase NAHD | Ps135530 | Pr72102 | Ha805196 | SPRG_06782 |
| Glutamate synthase Ferridoxin | Ps130831 | Pr78125 | Ha812981 | SPRG_06781 |
| Glutamate dehydrogenase | Ps108919 | Pr71959 | Ha805610 | SPRG_01659 |
| ATP sulfurylase  Adenylsulfate kinase Pyrophosphatase | Ps112102 | Pr79353 | Ha813786 | SPRG_02261 |
| Phosphoadenosine  phosphosulfate reductase | Ps156997 | Pr74880 | Ha809449 | SPRG_03629 |
| Sulfite reductase | Ps139488 | Pr71878 | none | none |
| Cysteine synthetase | Ps109175 | Pr71224 | Ha814750 | SPRG_09898 |

*****the absence of the orthologous gene encoding the enzymes are indicated by ‘none’ and shaded.
