# Supplementary material for: Distinctive Expansion of Potential Virulence Genes in the Genome of the Oomycete Fish Pathogen Saprolegnia parasitica
Source: PLoS Genet. 2013 Jun 13;9(6):e1003272. doi: 10.1371/journal.pgen.1003272 (PMC3681718; doi:10.1371/journal.pgen.1003272)
Supplement: Table S6 — Phospholipid modifying and signaling enzymes in Saprolegnia parasitica and other oomycetes. (DOCX) [file pgen.1003272.s018.docx]

**Supplemental Table S6– Phospholipid modifying and signaling enzymes in *Saprolegnia parasitica* and other oomycetes**

|  | *S.pa** | *P inf* | *Psoj* | *P ram* | *Hpa* | *Pult* |
| --- | --- | --- | --- | --- | --- | --- |
| PIS | 1 | 1 | 1 | 1 | 1 | 1 |
| PIK | 27 | 24 | 23 | 23 | 17 | 24 |
| PIPK | 16 | 16 | 16 | 16 | 15 | 16 |
| PLC | 1 | 0 | 0 | 0 | 0 | 0 |
| DGK | 2 | 1 | 1 | 1 | 1 | 1 |
| PLD | 8 | 18 | 18 | 18 | 9 | 9 |
| Total PMSE | 55 | 60 | 59 | 59 | 43 | 51 |

*The species names are Spa- *Saprolegnia parasitica,* Pinf *– Phytophthora infestans,* Psoj *– P. sojae,* Pram *– P. ramorum,* Hpa*– Hyaloperonospora Arabidopsidis,* Pult *– Pythium ultimum*.
