# Supplementary material for: Distinctive Expansion of Potential Virulence Genes in the Genome of the Oomycete Fish Pathogen Saprolegnia parasitica
Source: PLoS Genet. 2013 Jun 13;9(6):e1003272. doi: 10.1371/journal.pgen.1003272 (PMC3681718; doi:10.1371/journal.pgen.1003272)
Supplement: Table S7 — Unique and expanded domains in Saprolegnia parasitica proteome. (DOCX) [file pgen.1003272.s019.docx]

**Supplemental Table S7 - Unique and expanded domains in *Saprolegnia parasitica* proteome**

| pfam_ID# | pfam_name | Spa* | Pinf | Psoj | Pram | Hpa | Pult |
| --- | --- | --- | --- | --- | --- | --- | --- |
| PF00200 | Disintegrin | 16 | 0 | 0 | 0 | 0 | 0 |
| PF06109 | HlyE | 9 | 0 | 0 | 0 | 0 | 0 |
| PF02140 | Gal_Lectin | 5 | 0 | 0 | 0 | 0 | 0 |
| PF05257 | CHAP | 5 | 0 | 0 | 0 | 0 | 0 |
| PF03738 | GSP_synth | 4 | 0 | 0 | 0 | 0 | 0 |
| PF00722 | Glyco_hydro_16 | 3 | 0 | 0 | 0 | 0 | 0 |
| PF10067 | DUF2306 | 3 | 0 | 0 | 0 | 0 | 0 |
| PF04143 | DUF395 | 3 | 0 | 0 | 0 | 0 | 0 |
| PF03386 | ENOD93 | 3 | 0 | 0 | 0 | 0 | 0 |
| PF01384 | PHO4 | 3 | 0 | 0 | 0 | 0 | 0 |
| PF01373 | Glyco_hydro_14 | 2 | 0 | 0 | 0 | 0 | 0 |
| PF00728 | Glyco_hydro_20 | 2 | 0 | 0 | 0 | 0 | 0 |
| PF03051 | Peptidase_C1_2 | 2 | 0 | 0 | 0 | 0 | 0 |
| PF08142 | AARP2CN | 2 | 0 | 0 | 0 | 0 | 0 |
| PF00797 | Acetyltransf_2 | 2 | 0 | 0 | 0 | 0 | 0 |
| PF03960 | ArsC | 2 | 0 | 0 | 0 | 0 | 0 |
| PF07876 | Dabb | 2 | 0 | 0 | 0 | 0 | 0 |
| PF00684 | DnaJ_CXXCXGXG | 2 | 0 | 0 | 0 | 0 | 0 |
| PF07717 | DUF1605 | 2 | 0 | 0 | 0 | 0 | 0 |
| PF10019 | DUF2261 | 2 | 0 | 0 | 0 | 0 | 0 |
| PF04457 | DUF504 | 2 | 0 | 0 | 0 | 0 | 0 |
| PF05705 | DUF829 | 2 | 0 | 0 | 0 | 0 | 0 |
| PF03328 | HpcH_HpaI | 2 | 0 | 0 | 0 | 0 | 0 |
| PF04982 | HPP | 2 | 0 | 0 | 0 | 0 | 0 |
| PF08709 | Ins145_P3_rec | 2 | 0 | 0 | 0 | 0 | 0 |
| PF04212 | MIT | 2 | 0 | 0 | 0 | 0 | 0 |
| PF03348 | Serinc | 2 | 0 | 0 | 0 | 0 | 0 |
| PF03458 | UPF0126 | 2 | 0 | 0 | 0 | 0 | 0 |
| PF00023 | Ank | 286 | 167 | 181 | 133 | 56 | 140 |
| PF00520 | Ion_trans | 58 | 26 | 35 | 26 | 6 | 21 |
| PF00112 | Peptidase_C1 | 48 | 21 | 18 | 24 | 17 | 19 |
| PF01554 | MatE | 44 | 17 | 18 | 14 | 6 | 17 |
| PF00734 | CBM_1 | 40 | 4 | 5 | 5 | 0 | 3 |
| PF00652 | Ricin_B_lectin | 40 | 6 | 11 | 11 | 3 | 4 |
| PF03457 | HA | 36 | 4 | 8 | 4 | 2 | 2 |
| PF00415 | RCC1 | 31 | 32 | 12 | 11 | 13 | 14 |
| PF07714 | Pkinase_Tyr | 29 | 1 | 24 | 16 | 6 | 1 |
| PF00447 | HSF_DNA-bind | 28 | 8 | 11 | 10 | 6 | 6 |
| PF00375 | SDF | 27 | 6 | 8 | 10 | 4 | 0 |
| PF02214 | K_tetra | 26 | 3 | 2 | 1 | 1 | 1 |
| PF04547 | DUF590 | 21 | 8 | 11 | 10 | 6 | 9 |
| PF04749 | PLAC8 | 21 | 13 | 13 | 17 | 2 | 15 |
| PF02798 | GST_N | 19 | 4 | 11 | 5 | 5 | 11 |
| PF01266 | DAO | 17 | 6 | 10 | 7 | 6 | 6 |
| PF07974 | EGF_2 | 17 | 7 | 13 | 10 | 3 | 15 |
| PF01757 | Acyl_transf_3 | 16 | 7 | 14 | 10 | 3 | 5 |
| PF00753 | Lactamase_B | 16 | 8 | 8 | 6 | 7 | 6 |
| PF01764 | Lipase_3 | 15 | 4 | 7 | 4 | 5 | 5 |
| PF00066 | Notch | 15 | 1 | 1 | 1 | 1 | 1 |
| PF04564 | U-box | 15 | 2 | 4 | 2 | 2 | 3 |
| PF00650 | CRAL_TRIO | 14 | 6 | 6 | 6 | 4 | 4 |
| PF01485 | IBR | 14 | 2 | 1 | 3 | 3 | 4 |
| PF00566 | TBC | 14 | 6 | 6 | 5 | 4 | 8 |
| PF01145 | Band_7 | 13 | 7 | 9 | 6 | 3 | 7 |
| PF04116 | FA_hydroxylase | 13 | 2 | 3 | 3 | 2 | 3 |
| PF00704 | Glyco_hydro_18 | 12 | 2 | 3 | 2 | 2 | 2 |
| PF00246 | Peptidase_M14 | 11 | 6 | 4 | 6 | 2 | 8 |
| PF05577 | Peptidase_S28 | 10 | 5 | 4 | 4 | 2 | 5 |
| PF00533 | BRCT | 9 | 9 | 4 | 3 | 3 | 4 |
| PF00903 | Glyoxalase | 9 | 1 | 1 | 1 | 1 | 1 |
| PF01544 | CorA | 8 | 3 | 5 | 4 | 4 | 1 |
| PF01436 | NHL | 8 | 4 | 2 | 1 | 0 | 2 |
| PF00144 | Beta-lactamase | 7 | 1 | 2 | 2 | 1 | 2 |
| PF01432 | Peptidase_M3 | 7 | 3 | 3 | 3 | 2 | 3 |
| PF01612 | 3_5_exonuc | 7 | 2 | 2 | 2 | 3 | 3 |
| PF01221 | Dynein_light | 7 | 4 | 1 | 5 | 1 | 6 |
| PF00042 | Globin | 7 | 1 | 2 | 2 | 0 | 2 |
| PF01644 | Chitin_synth_1 | 6 | 1 | 1 | 2 | 1 | 1 |
| PF01223 | Endonuclease_NS | 6 | 1 | 1 | 1 | 1 | 1 |
| PF07728 | AAA_5 | 6 | 1 | 4 | 4 | 1 | 3 |
| PF00857 | Isochorismatase | 6 | 0 | 1 | 1 | 0 | 1 |
| PF09286 | Pro-kuma_activ | 6 | 2 | 2 | 2 | 2 | 1 |
| PF01636 | APH | 5 | 1 | 1 | 1 | 2 | 1 |
| PF00635 | Motile_Sperm | 5 | 3 | 2 | 3 | 2 | 2 |
| PF05089 | NAGLU | 5 | 2 | 2 | 2 | 0 | 1 |
| PF00883 | Peptidase_M17 | 4 | 2 | 2 | 2 | 2 | 2 |
| PF03358 | FMN_red | 4 | 1 | 0 | 1 | 0 | 0 |
| PF05183 | RdRP | 4 | 0 | 0 | 0 | 1 | 0 |
| PF00092 | VWA | 4 | 3 | 2 | 5 | 1 | 1 |
| PF00244 | 14-3-3 | 3 | 1 | 1 | 1 | 1 | 1 |
| PF03381 | CDC50 | 3 | 1 | 1 | 1 | 2 | 1 |
| PF03781 | DUF323 | 3 | 3 | 1 | 1 | 0 | 0 |

*The species names are Spa- *Saprolegnia parasitica,* Pinf *– Phytophthora infestans,* Psoj *– P. sojae,* Pram *– P. ramorum,* Hpa*– Hyaloperonospora Arabidopsidis,* Pult *– Pythium ultimum*.

#The domains in *S. parasitica* occurring more than twice as that of the average plant oomycete pathogens and having more than 3 members are considered to be enriched.
